# Maternal and paternal depressive symptoms and parental vocalisation behaviours in infancy: findings from UK-based birth cohort

**DOI:** 10.3389/frcha.2023.1122371

**Published:** 2023-06-19

**Authors:** Amy Campbell, Gemma Lewis, Ilaria Costantini, Miguel Cordero, Andy Skinner, Esther Dermott, Tina Miller, Mari-Rose Kennedy, Iryna Culpin

**Affiliations:** ^1^ MRC Integrative Epidemiology Unit, University of Bristol, Bristol, United Kingdom; ^2^ School of Psychological Science, University of Bristol, Bristol, United Kingdom; ^3^ Centre for Academic Mental Health, University of Bristol, Bristol, United Kingdom; ^4^ Division of Psychiatry, University College London, London, United Kingdom; ^5^ Faculty of Medicine, Universidad del Desarollo, Santiago, Chile; ^6^ Population Health Sciences, University of Bristol, Bristol, United Kingdom; ^7^ School for Policy Studies, University of Bristol, Bristol, United Kingdom; ^8^ School of Law and Social Sciences, Oxford Brookes University, Oxford, United Kingdom; ^9^ Department of Psychology, Manchester Metropolitan University, Manchester, United Kingdom

**Keywords:** Avon Longitudinal Study of Parents and Children (ALSPAC), paternal postnatal depression, maternal postnatal depression, parental vocalisation, infant vocalisation, parent-infant interactions, behavioural micro-coding, birth-cohort study

## Abstract

**Background:**

Both maternal and paternal postnatal depression (PND) are associated with increased risk of less optimal offspring developmental outcomes. Early exposure to differences in maternal and paternal vocalisation behaviours associated with maternal and paternal PND may be important in this relationship. However, little research has captured vocalisation patterns at home without researchers present.

**Objectives:**

This study sought to examine the associations between maternal and paternal PND and various aspects of parental vocalisation behaviours.

**Methods:**

Mothers (*n* = 104) and fathers (*n* = 34) of six-months old infants from the Avon Longitudinal Study of Parents and Children Generation-2 (ALSPAC-G2) provided video footage of mother- and father-infant interactions filmed at home using the head-worn video cameras (headcams) without the need for researchers to be present. Twenty-five mother-infant and father-infant interactions were coded on multiple aspects of parental and infant vocalisation behaviours using the micro-behavioural observational coding system. Parental (PND) was measured using the Edinburgh Postnatal Depression Scale (EPDS; total score).

**Results:**

Frequencies and duration of vocalisation behaviours were similar in mothers and fathers. However, there was an indication that fathers demonstrated higher frequency and duration of commands, exclamations and ironic/sarcastic tone, and criticisms compared to mothers, while mothers engaged in more teaching compared to fathers. Linear regression models indicated that maternal and paternal PND were not associated with the majority of vocalisation behaviours. However, there were some specific patterns observed, mostly related to the emotional tone of the vocalisations. Higher levels of maternal PND were associated with lower frequency of speech in a neutral tone, frequency and duration of use of humour, and increased duration of speech in a positive tone. Higher levels of paternal PND were associated with higher mean duration of speech, infant-directed speech, higher frequency and duration of laughing, and increased duration of speech using questions and encouragement.

**Conclusion:**

These findings extend existing research by investigating the associations between maternal and paternal PND and a wide range of vocalisation behaviours captured and coded using innovative methods and in a more ecologically valid way than previous studies.

## Introduction

A large body of research has now documented the high prevalence of maternal postnatal depression [PND; (1)], its link to potentially negative offspring development (2), and associated health and societal consequences (3). Despite mounting evidence to support increased risk of postnatal depression in men [PND; (1)], and associated adverse offspring outcomes (4), the epidemiology of paternal PND and its impact on the child has received less attention in research. The overall prevalence of maternal postnatal depression has been estimated at 23.8%, while in men this estimate approximates 10.4% (1) with several studies reporting strong associations between maternal and paternal mood during this period (1, 5, 6).

Many studies have shown that parenting behaviour is less sensitive and attuned in the context of maternal PND (7–9), which is on the of the potential pathways for mental health risk transmission in families (10). In comparison, pathways from paternal PND to offspring outcomes are less well understood, but paternal parenting behaviours are also emerging as a potential pathway of transmission (11). A meta-analysis of 28 observational studies concluded that paternal PND has small but statistically significant effects on parenting. Fathers who experienced depression demonstrated small, but meaningful, decreases in positive parenting behaviours (*r* = −0.19; e.g., less warmth, sensitivity, and appropriate discipline) and small, but meaningful, increases in negative parenting behaviours (*r* = 0.16; e.g., more hostility, intrusiveness, and inappropriate discipline). These effect sizes were comparable to the associations found between PND and maternal positive (*r* = −0.20) and negative parenting (*r* = 0.22) (11).

Traditionally, broad positive and negative domains of maternal (9) and paternal (11) parenting in the context of parental depression have been investigated. However, more insights into the specific manifestations of these broad constructs are needed to improve the identification of potential intervention targets (12, 13). Specifically, maternal vocalisations are one of the possible manifestations of impaired responsiveness that characterise mother-infant interactions in the context of depression (14), which have also been linked to adverse offspring outcomes (15). Similarly, one of the key pathways through which paternal PND may influence offspring development is through its effects on father-child interactions, including paternal vocalisation behaviours (11).

Both maternal and paternal vocalisation behaviours are important for a range of offspring outcomes, including language (16, 17), socio-emotional and cognitive development (16, 18, 19). For instance, frequency of maternal vocalisation has been found to be positively associated with cognitive development and educational attainment (20, 21) and negatively associated with childhood psychopathology (22). Furthermore, the quality of maternal vocal interaction with her infant has been shown to be more important for child language outcomes than global maternal sensitivity [defined as maternal ability to perceive and to interpret the signals of her child, and promptly and appropriately respond to them; (7, 23)]. There is also evidence that mothers who experience depression vocalise less when interacting with their infants (24), are less likely to increase the mean length of their utterances as their children develop (25), and use fewer words overall when interacting with their infants (26). Mothers with depression are also less likely to use specific types of vocalisations associated with better child outcomes. It has been shown, albeit inconsistently (27), that the frequency of infant-directed speech [characterised by a sing-song pitch, generally carried by exaggerated prosody compared to the more monotone style used to communicate with adults; (28)] decreases as maternal PND increases (29). Although mothers with depression tend to speak at an overall higher mean pitch when addressing their infants (25), the infant-directed speech becomes flatter, and more restricted (30, 31), which may be interpreted by infants as less positive (32) and have a negative impact on infant associative learning (33, 34). A recent systematic review found evidence for a reduction in the amount, but not the complexity, of infant-directed speech as maternal depressive symptoms increased (35). It is important to note that these studies may not capture culture-specific variations in depressed mothers' speech (36). Mind-minded speech (defined as caregiver's ability to comment appropriately on infant's putative internal states during interactions) is an important part of the parental vocalization repertoire (37), which reflects parental ability to accurately recognise infant's mental state (38). Maternal PND has been found to be associated with lower frequency of mind-minded speech in a clinical sample (39), with maternal ability to recognise their infant's agency also being impaired (20). In addition, mothers with depression have been found to use more self-focused speech (40), which is more likely to contain words with negative valence, criticism, and/or hostility (20, 41).

Unlike research on maternal PND and vocalisation behaviours, there remains a paucity of studies that examine the impact of paternal PND on different aspects of paternal vocalisations (19, 42). The few previous studies on vocalisation in fathers with depression have focused on specific aspects, such as parental speech registers with pre-verbal infants (43) and cognitive and mentalising features (19), rather than a comprehensive range of vocalisation behaviours. Compared to fathers without PND, fathers with PND use speech that is more focused on paternal rather than infants' experiences, comprising more negative and critical utterances (19), as well as being lower in modulation (43).

It should be noted that important differences between mothers and fathers have been noted in the literature on wider aspects of parent-infant interactions, including the more physically arousing nature of paternal compared to maternal play (44). However, a more recent comparison of studies between maternal and paternal vocalisation behaviours suggests that the similarities seem to outweigh the differences during infancy, with both mothers and fathers modifying their speech when interacting with their infants (45). It has also been suggested that depression has a comparable effect on parenting and parent-child interactions, with both mothers and fathers who experience PND displaying increases in hostility, instrusiveness, and disengagement, and decreases in sensitivity, warmth and responsiveness (11). Limited existing research precludes any conclusions as to whether any such similarities or differences map onto depressed mothers' and fathers’ vocalisation behaviours with their infants.

The validity and reliability of measures to assess parenting is a key issue in developmental research (46, 47). Both self-reported and independently observed measures of parenting are subject to limitations, including reporting (48–50) and social desirability biases (51). Despite the limitations, observational measures are better predictors of offspring cognitive and emotional outcomes (51) and are particularly sensitive to detect changes in parental and infant behaviours following interventions (46) compared to self-reported measures. Social desirability bias may be addressed by using more ecologically valid first-person cameras [hereafter referred to as headcams; see (52) for detailed description], which reduce demand characteristics whilst capturing a higher frequency of less socially desirable maternal behaviours compared to “gold standard” observational methods [i.e., researcher observing or filming the interaction in a clinical, research or home setting; (52)].

The aims of the current study were to examine the associations between both maternal and paternal PND and a comprehensive range of infant-directed maternal and paternal vocalisation behaviours. Vocalisation behaviours were recorded in the home without the presence of a researcher using the headcams, maximising the possibility of capturing naturalistic interactions and reducing demand characteristics (52). Four vocalisation behaviours of interest were identified *a priori*—parental speech, infant-directed and mind-minded speech, and emotional tone of speech. As existing research on the associations between paternal PND and vocalisations is limited, these behaviours were selected, and hypotheses were made, based on studies of maternal vocalisations. Specifically, we hypothesised that maternal and paternal PND would be associated with (1) lower frequency and duration of all speech, infant-directed and mind-minded speech, as well as positive emotional tone; and (2) higher frequency and duration of negative and neutral emotional tone. We examined the associations between maternal and paternal depression and all other vocalisation behaviours in a hypothesis-free manner.

## Methods

### Study cohort

**Figure 1:**
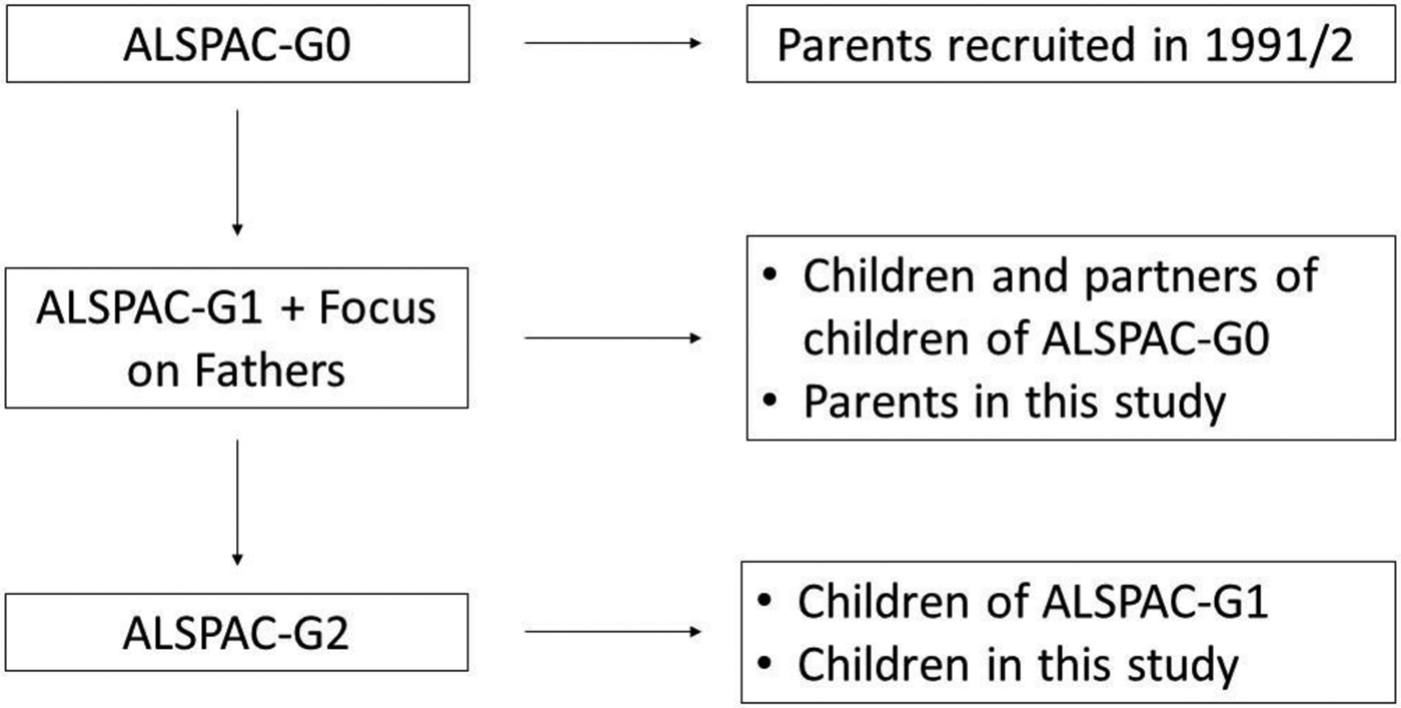
Flow chart of ALSPAC structure in relation to this study.

The sample comprised participants from the ALSPAC cohort (Figure 1). During Phase I enrolment, 14,541 pregnant mothers residing in the former Avon Health Authority in the south-west of England with expected dates of delivery between 1 April 1991 and 31 December 1992 were recruited. The total sample size is 15,454 pregnancies, of which 14,901 were alive at 1 year of age. ALSPAC Generation 2 (ALSPAC-G2) was set up to provide a unique multigenerational cohort and builds on the existing ALSPAC resource of originally recruited women and their partners (Generation 0; ALSPAC-G0) and their offspring (ALSPAC-G1) followed up for 26 years. Recruitment of the next generation ALSPAC-G2—the grandchildren of ALSPAC-G0 and children of ALSPAC-G1—began on 6th June 2012. Up to 30th June 2018, 810 ALSPAC-G2 participants from 548 families had been recruited. Over 70% of those invited to early- and late-pregnancy, second week of life, 6-, 12- and 24-month assessments attended, with attendance >60% for subsequent visits up to 7 years. Further details on the cohort profile, representativeness and phases of recruitment, including ALSPAC-G2, are described in four cohort-profile papers (53–56). ALSPAC study website www.bristol.ac.uk/alspac/ contains details of all the data that is available through a fully searchable data dictionary and variable search tool (http://www.bris.ac.uk/alspac/researchers/our-data/).

### Ethical approval

Informed consent for the use of data collected via questionnaires and clinics was obtained from participants following the recommendations of the ALSPAC Ethics and Law Committee at the time. Study data were collected and managed using Research Electronic Data Capture (REDCap), a secure web-based electronic data capture tools hosted at the University of Bristol (57).

### Recruitment into the headcams study

A wide range of social, lifestyle, clinical and biological data have been collected on all family members repeatedly, including videos of parent-child interactions recorded using the headcams. Recruitment of mothers into the headcams study began on 7th July 2016, with 422 (90%) of mothers and their infants attending a 6-months assessment at the research clinic. 266 (63%) of mothers who attended the clinic were invited to record interactions with their infant using the headcams at home. 141 (53%) of these mothers consented to participate and 104 (74%) mothers provided video footage of mother-infant interactions. Initially, biological fathers and mothers' partners were invited to participate in the headcams study indirectly through an invitation to the mother when their child joined ALSPAC-G2. On 22nd July 2019, through additional funding from Wellcome Trust, a separate research clinic for fathers was set up (Focus on Fathers) inviting fathers directly to attend a range of assessments, including the headcams, when their G2 child was six months old. Overall, 194 fathers were invited to attend, with 83 (43%) fathers consenting to participate and 34 (18%) fathers providing video footage of father-infant interactions. For the purposes of this study, 25 mother-infant and 25 father-infant dyads were fully coded on various aspects of parental and infant vocalisations using the micro-behavioural observation coding system (58).

### Videorecording procedures using the headcams

We captured video and audio footage of mother- and father-infant interactions using the headcams previously used for recording infant's eye view of their environment (59). The headcams are worn on headbands by both the parent (mother and father) and the infant, capturing two separate videos from the parent and infant perspective for each interaction. Headcams have previously been shown to be reliable for capturing mother and infant behaviours (52) and have been extensively used with fathers in the ALSPAC cohort. A questionnaire enquiring about fathers' experiences of using the headcams suggested that fathers perceive them to be user-friendly with no bearing on how they or their infant engage in the interaction. Separate headcam footage from both the parent and infant cameras were synchronised by the researchers for coding purposes. Headcam protocols were identical for both mothers and fathers. Parents were given fully-charged headcams and asked to use them at home during mealtime and play interactions. For the mothers, interactions analysed in this study were classed as “mealtime” (infant engages in eating = 24) and “stacking task” (mother and infant engage in a play task with a stacking toy; *n* = 1). For fathers, the interactions were classed as “mealtime” (*n* = 15); “stacking task” (*n* = 4), and “free play task” (father and infant engage in a play as they normally would; *n* = 6). Examples of each activity are presented in the MHINT micro-coding scheme (58). All videos were recorded at participants' home. Thus, it was possible for siblings/other caregivers/pets to be present during the interactions. Only three out of 25 children were from the same mother-father-child triad. Videos for 25 distinct mother-infant, and 25 distinct father-infant dyads (one father provided two interactions) were used in the present analyses.

### Measures

#### Exposures: maternal and paternal postnatal depression (PND)

We refer to parental PND not as a clinical diagnosis, but as experiences of self-reported maternal and paternal depressive assessed using the Edinburgh Postnatal Depression Scale [EPDS; (60)], a 10-item self-reported questionnaire validated with women and men for use during the perinatal period (61, 62). Maternal depressive symptoms were assessed at multiple time points, including early (<20 weeks) and late pregnancy (>28 weeks), and 715 days after birth with the measure completed closest to the six-month assessment included in the analyses. Paternal depressive symptoms were extracted from birth, annual and six months postnatal questionnaires with all measures included in the analyses to maximise the sample (none of the fathers had EPDS measures available at all three time points). In order to capture the full variation in depressive symptoms, individual depression items were summed to derive a continuous score used in all analyses (score range: mothers 0–30; fathers 0–24 with higher scores indicating more severe depressive symptoms).

#### Outcome: parental and infant vocalisation behaviours

**Figure 2:**
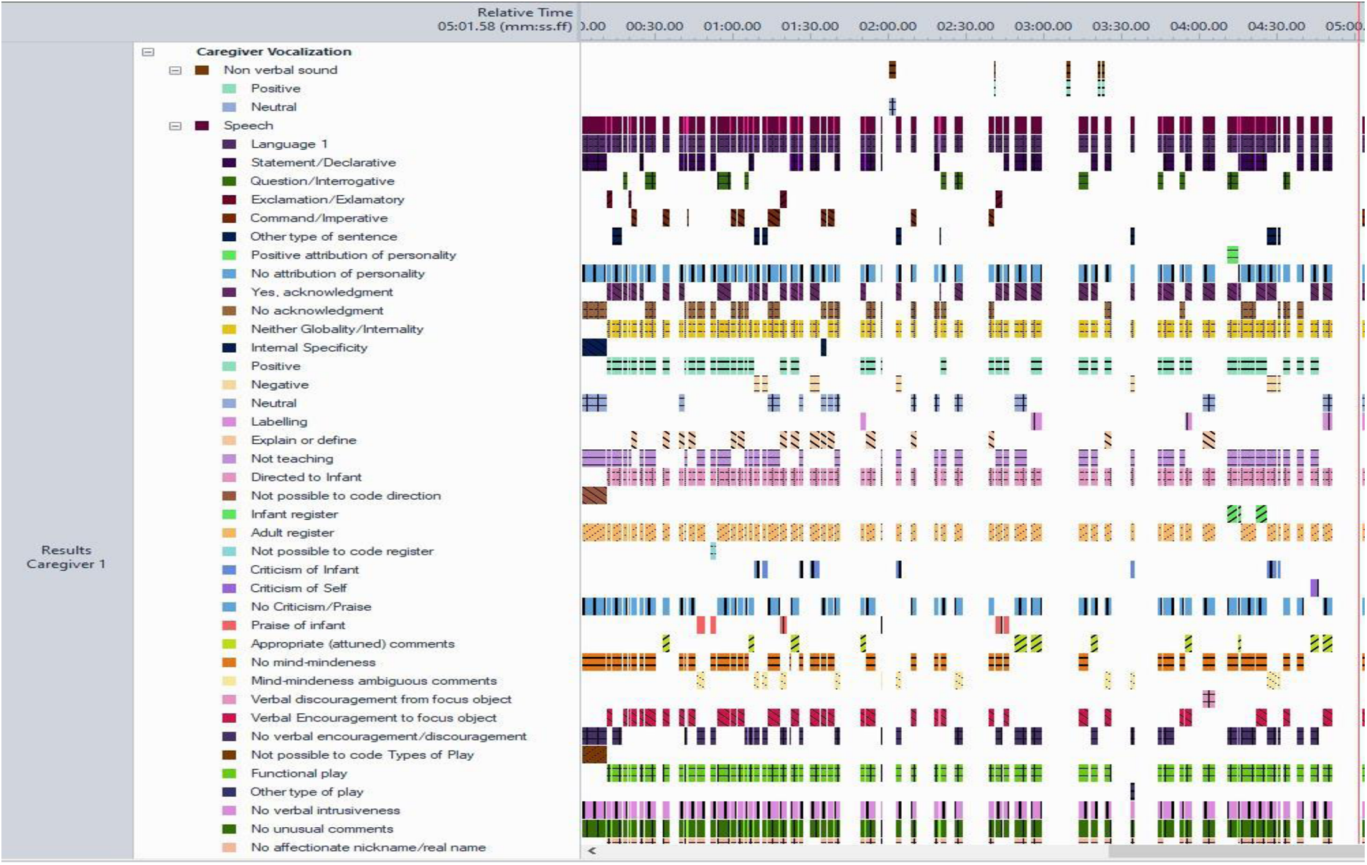
Example of paternal vocalisation behaviours visualised using behavioural software observer-XT (length of video 5 min).

All interactions were coded on a continuous event-basis using the MHINT micro-coding scheme (58) and specialised software for behavioural research Noldus Observer XT 14.0 (64). In summary, within each behavioural group (e.g., caregiver vocalisation), behaviours (e.g., speech, laugh) are mutually exclusive and exhaustive, thus, at each point in time, exactly one behaviour from each behavioural group must be coded. Modifiers allow for more detailed categorisation within a behavioural group (e.g., within “vocalisation” behavioural group, modifiers allow the coder to categorise the tone of the speech as positive, negative, or neutral). If a modifier is associated with a behavioural group, both the behaviour group and the modifier group must be coded. For the purposes of this study, only parental and infant vocalisations were coded, which included any sound made by the infant and caregiver – voluntary or involuntary and meaningful (i.e. any sound which is not verbal but still has a communicative meaning; e.g., sighs) or non-meaningful (e.g., yawning) sounds. Within “caregiver vocalisation” behavioural group, there are seven behaviours and twenty-two potential modifiers, while “infant vocalisation” behavioural group is composed of nine behaviours and five modifiers (Table 1). The full manual describing the micro-behavioural observational coding system is available online, including an exhaustive list of all overarching behavioural codes, individual behaviours and modifiers (58). For visualisation of codes underlying parental vocalisation behaviours please see Figure 2.

**Table 1:** Caregiver and infant vocalisation, corresponding behaviours and modifiers.

Behavioural group^a^	Behaviour	Modifiers^b^
Caregiver vocalisation	Speech	Language
		Type of sentence
		Tone
		Infant- and adult-directed speech
		To whom speech is directed
		Acknowledgement
		Reference to self
		Attribution of personality
		Criticism
		Praise
		Use of humour
		Attuned and non-attuned mind-mindedness (63)
		Encouragement
		Discouragement
		Verbal intrusiveness
		Verbal role reversal
		Verbal play
		Odd content
		Sociality of verbal play
		Globality and specificity
		Teaching
		Use of real name/nickname
	Musical sounds	–
	Laugh	Nervous laugh
	Vocal imitation	Tone
	Bodily sounds	Type of bodily sound
	Non-verbal sounds	Tone
	Silent	–
	NPTC caregiver vocalisation	–
Infant vocalisation	Laughing	–
	Distressed	–
	Non-distressed	Tone
	Vocal imitation	Tone
	Babbling	Tone
	First words	Tone
		First words type
		Type of sentence
		Verbal role reversal
	Screaming	Tone
	Bodily sounds	Type of bodily sounds
	Silent/none of the above	–
	NPTC infant vocalisation	–

NPTC, not possible to code.

^a^ Behavioural group refers to an overarching behavioural category, comprised of mutually exclusive and exhaustive behaviours.

^b^ Modifiers allow for more detailed categorisation within behavioural group.

Videos of maternal and paternal interactions were coded by different researchers. Coding of maternal interactions was completed by three independent female researchers with at least a Master's level qualification in psychology or a related discipline, all of whom are co-authors on this paper. Coding of paternal interactions was completed by one male and three female researchers with Master's and Doctorate qualifications in Psychology. Coders were trained in using the coding scheme and blind to parental depression status. For reliability purposes, four videos of mother-infant and four videos of father-infant interactions were double coded, with inter-rater reliability assessed using Cohen's kappa separately for the overall behaviour group (*κ* = 0.91; 0.90–0.93). All reliability analyses were conducted using Noldus Observer XT 14.0 (64). All videos were coded for five minutes in line with previous research (65) and recent evidence suggesting that thin slice sampling (i.e., <5 min) is a suitable approach across different behavioural groups, including vocalisation (66).

### Potential confounders

#### Maternal, paternal and infant characteristics

Analyses were adjusted for risk factors that have been previously found to be associated with maternal and paternal PND and parental vocalisation, including child sex (20), birth order [first born vs. second or later born; (67)], maternal (assessed at delivery) and paternal (assessed at six-months assessment) age in years (68) and parental highest educational attainment [compulsory secondary level up to age 16 years/GCSE vs. noncompulsory secondary level up to age 18 years/AS/A Levels and university level education/Degree and higher; (69)] assessed at birth.

#### Characteristics of mother- and father-infant interactions

Analyses were also adjusted for a number of characteristics pertaining to mother- and father-infant interactions. Infants are active participants in the interaction and their behaviour may elicit certain behavioural responses in mothers (e.g., greater vocalisation in infant may evoke greater vocalisation in mother); thus, we adjusted for the frequency/duration of infant vocalisation. In addition, parent-child interactions were recorded at home, often with other caregivers/siblings present. Therefore, we adjusted for the total duration of caregiver speech directed solely at the infant.

### Statistical analyses

First, we examined characteristics of our sample by parental and child characteristics (Table 2) and frequency and duration of parental and infant vocalisation behaviours (Table 3) using *χ*^2^ and ANOVA tests. We ran Pearson correlations to examine the associations between maternal and paternal PND and infant vocalisations. Second, we conducted linear regressions to examine the associations between maternal and paternal PND (continuous exposures) and the frequency (mean rate per minute) and mean duration of parental vocalisation behaviours as continuous outcomes. Parental vocalisations that violated the assumptions of linear regression or were uncommon (e.g., criticism, intrusiveness, discouragement) were either dichotomised using a median split or divided into equal quintiles to derive ordinal variables. We then conducted ordinal logistic regressions and logistic regressions to examine the odds of being in a lower quintile or a category with a lower frequency or mean duration of each vocalisation behaviour as parental PND score increased. All models were first estimated unadjusted (exposure and outcome only), following incremental adjustment for amount of speech directed to infant, frequency/mean RPM/mean duration of infant vocalisation, and parental and child characteristics. Hypotheses-led analyses included models examining associations between maternal and paternal depression and frequency and duration of parental (1) all speech; (2) infant-directed speech; (3) attuned mind-mindedness; (4) positive emotional tone; (5) negative emotional tone; and (6) neutral emotional tone. The associations between maternal and paternal PND and the frequency and duration of all other parental vocalisation behaviours were examined without specific hypotheses. All analyses were conducted in Stata v.15 (Stata Corp., Texas, USA) (70) A sensitivity power analysis conducted using G*Power 3.1 (71) indicated that with a sample size of 25, *α* = 0.05, and 80% power, the minimum effect sizes this study was powered to detect were *β* = 0.51 and POR = 3.56. However, given that multiple tests were conducted, effect sizes at and above these thresholds could still be underpowered and findings were interpreted with caution.

**Table 2:** Characteristics of the study sample.

	Parental characteristics	
	Fathers	Mothers
Parental education, *n* (%)		
GCSE	14 (56%)	6 (24%)
AS/A level	7 (28%)	13 (52%)
Degree/higher	<5 (–)^*^	5 (20%)
Birth order, *n* (%)		
First-born	19 (76%)	17 (68%)
Second or later born	6 (24%)	8 (32%)
Parental age, mean (SD)	28.8 (3.58)	25.0 (1.06)
Parental PND (EPDS), mean (SD)	8.17 (5.11)	6.61 (5.37)
	Child characteristics	
	Boys	Girls
Child gender, *n* (%)	23 (46%)	27 (54%)
Child age, mean (SD)	30.9 (3.74)	30.4 (7.89)

EPDS, Edinburgh Postnatal Depression Scale; PND, postnatal depression.

^*^ Censored to prevent disclosure because of small cell counts.

**Table 3:** Frequency and duration of parental and infant vocalisation behaviours.

Vocalisations	Infant vocalisation behaviours					
	*N* (%)		Mean RPM (SD)		Mean duration (SD)	
	Fathers	Mothers	Fathers	Mothers	Fathers	Mothers
**Distress**	15 (60%)	15 (60%)	0.80 (1.32)	0.69 (0.95)	5.40 (9.84)	2.67 (3.95)
*χ*^2^ and ANOVA, *p*-value		0.00, 1.00		0.35, 0.724		1.29, 0.204
**Non-distress**	24 (96%)	23 (92%)	1.87 (1.46)	2.74 (2.40)	3.77 (3.71)	2.14 (2.11)
*χ*^2^ and ANOVA, *p*-value		0.35, 0.552		−1.53, 0.131		1.90, 0.062
**Any vocalisation^a^**	25 (100%)	24 (96%)	1.02 (0.73)	1.35 (1.00)	5.42 (5.47)	5.33 (8.93)
*χ*^2^ and ANOVA, *p*-value		1.02, 0.312		−1.35, 0.184		0.05, 0.963
	**Parental vocalisation behaviours**					
**Laughter**	17 (68%)	17 (68%)	0.46 (0.55)	0.36 (0.41)	1.42 (1.36)	1.23 (1.55)
*χ*^2^ and ANOVA, *p*-value		0.00, 1.00		0.76, 0.449		0.45, 0.654
**Vocal imitation** ^*^	<5 (–)^*^	5 (20%)	0.02 (0.12)	0.14 (0.40)	0.08 (0.40)	0.37 (0.91)
*χ*^2^ and ANOVA, *p*-value		3.03, 0.082		−1.43, 0.16		−1.46, 0.150
**Musical sounds** ^*^	5 (20%)	7 (28%)	0.15 (0.45)	0.20 (0.50)	0.49 (1.09)	2.33 (5.66)
*χ*^2^ and ANOVA, *p*-value		0.44, 0.508		−0.36, 0.718		−1.60, 0.116
**Speech**	25 (100%)	25 (100%)	7.71 (3.01)	7.09 (3.50)	2.20 (0.62)	2.47 (2.47)
*χ*^2^ and ANOVA, *p*-value		–		0.67, 0.503		−0.89, 0.376
**Non-verbal sounds**	22 (88%)	24 (96%)	1.37 (1.16)	1.45 (1.29)	1.79 (1.14)	1.49 (0.96)
*χ*^2^ and ANOVA, *p*-value		1.42, 0.492		−0.23, 0.819		0.98, 0.327
**Silence**	25 (100%)	23 (92%)	6.54 (2.17)	6.89 (4.11)	7.46 (8.79)	5.50 (3.72)
*χ*^2^ and ANOVA, *p*-value		2.08, 0.149		−0.38, 0.707		1.03, 0.309
	**Modifiers of parental speech**					
**Command**	23 (92%)	14 (56%)	0.82 (0.69)	0.45 (0.63)	1.77 (0.71)	1.34 (1.77)
*χ*^2^ and ANOVA, *p*-value		8.42, 0.004		1.94, 0.058		1.14, 0.261
**Exclamation**	20 (80%)	10 (40%)	0.58 (0.54)	0.20 (0.30)	1.96 (1.21)	1.17 (3.16)
*χ*^2^ and ANOVA, *p*-value		10.70, 0.005		3.13, 0.003		1.16, 0.253
**Question**	24 (96%)	24 (96%)	2.01 (1.08)	2.13 (1.50)	2.00 (0.84)	2.43 (2.15)
*χ*^2^ and ANOVA, *p*-value		0.00, 1.00		−0.34, 0.732		−0.93, 0.355
**Statement**	25 (100%)	25 (100%)	2.86 (1.64)	3.08 (1.57)	2.70 (1.16)	2.74 (1.17)
*χ*^2^ and ANOVA, *p*-value		–		−0.48, 0.629		−0.11, 0.908
**Acknowledgement**	25 (100%)	25 (100%)	3.16 (1.59)	4.40 (3.56)	2.14 (0.68)	2.42 (1.66)
*χ*^2^ and ANOVA, *p*-value		–		−1.59, 0.118		−0.80, 0.425
**Neutral tone**	24 (96%)	25 (100%)	2.20 (1.53)	2.36 (1.28)	2.09 (0.81)	2.84 (1.52)
*χ*^2^ and ANOVA, *p*-value		1.02, 0.312		−0.40, 0.687		−2.16, 0.036
**Positive tone**	25 (100%)	25 (100%)	5.29 (2.72)	4.35 (3.50)	2.21 (0.76)	2.44 (1.44)
*χ*^2^ and ANOVA, *p*-value		–		1.06, 0.292		−0.72, 0.473
**Negative tone**	<5 (–)^*^	5 (20%)	0.13 (0.35)	0.03 (0.07)	0.58 (1.25)	0.21 (0.50)
*χ*^2^ and ANOVA, *p*-value		0.13, 0.713		1.34, 0.186		1.37, 0.177
**Ironic/Sarcastic tone**	8 (32%)	<5 (–)^*^	0.09 (0.14)	0.06 (0.15)	0.77 (1.28)	0.58 (1.81)
*χ*^2^ and ANOVA, *p*-value		1.75, 0.185		0.71, 0.480		0.41, 0.681
**Adult-directed speech**	25 (100%)	25 (100%)	5.84 (2.97)	3.60 (2.44)	2.2672	2.6632
*χ*^2^ and ANOVA, *p*-value		–		2.92, 0.005		−1.25, 0.218
**Infant-directed speech**	24 (96%)	25 (100%)	1.87 (1.81)	3.22 (3.07)	2.06 (1.14)	2.49 (1.60)
*χ*^2^ and ANOVA, *p*-value		1.02, 0.312		−1.88, 0.065		−1.09, 0.280
**Attribution of personality**	6 (24%)	9 (36%)	0.17 (0.45)	0.12 (0.24)	0.55 (1.18)	0.88 (1.63)
*χ*^2^ and ANOVA, *p*-value		0.86, 0.355		0.54, 0.592		−0.82, 0.415
**Praise of infant**	20 (80%)	16 (64%)	1.03 (1.31)	0.53 (0.98)	1.57 (0.90)	1.86 (2.69)
*χ*^2^ and ANOVA, *p*-value		1.59, 0.208		1.52, 0.134		−0.51, 0.609
**Praise of other person**	5 (20%)	<5 (–)^*^	0.04 (0.08)	0.04 (0.13)	0.95 (2.50)	0.25 (0.83)
*χ*^2^ and ANOVA, *p*-value		0.59, 0.440		−0.001, 0.999		1.33, 0.190
**Criticism of infant**	10 (40%)	<5 (–)^*^	0.19 (0.35)	0.03 (0.12)	0.98 (1.30)	0.29 (1.21)
*χ*^2^ and ANOVA, *p*-value		7.02, 0.008		2.12, 0.039		1.94, 0.057
**Criticism of other person** ^*^	5 (20%)	<5 (–)^*^	0.10 (0.33)	0.02 (0.06)	0.67 (1.74)	0.19 (0.62)
*χ*^2^ and ANOVA, *p*-value		0.59, 0.440		1.19, 0.239		1.31, 0.195
**Use of humour**	7 (28%)	12 (48%)	0.15 (0.32)	0.18 (0.26)	0.93 (1.69)	1.01 (1.31)
*χ*^2^ and ANOVA, *p*-value		2.12, 0.145		−0.38, 0.706		−0.17, 0.866
**Attuned mind-mindedness**	20 (80%)	13 (52%)	0.91 (1.20)	0.21 (0.26)	1.86 (1.16)	1.21 (1.66)
*χ*^2^ and ANOVA, *p*-value		4.37, 0.037		2.87, 0.006		1.62, 0.112
**Encouragement**	25 (100%)	23 (92%)	2.37 (2.41)	1.83 (1.75)	2.33 (1.65)	2.05 (1.32)
*χ*^2^ and ANOVA, *p*-value		2.08, 0.149		0.90, 0.371		0.66, 0.511
**Discouragement**	13 (53%)	14 (56%)	0.17 (0.21)	0.35 (0.52)	1.18 (1.35)	1.43 (2.44)
*χ*^2^ and ANOVA, *p*-value		0.08, 0.777		−1.65, 0.106		−0.44, 0.660
**Intrusiveness**	14 (56%)	<5 (–)^*^	0.68 (2.05)	0.12 (0.37)	1.71 (1.89)	0.31 (0.83)
*χ*^2^ and ANOVA, *p*-value		8.68, 0.003		1.35, 0.183		3.39, 0.001
**Role reversal** ^*^	<5 (–)^*^	<5 (–)^*^	0.01 (0.04)	0.06 (0.19)	0.08 (0.38)	0.38 (1.19)
*χ*^2^ and ANOVA, *p*-value		1.09, 0.297		−1.38, 0.173		−1.18, 0.243
**Verbal play**	15 (60%)	7 (28%)	2.09 (3.17)	0.14 (0.40)	1.33 (1.28)	0.65 (1.32)
*χ*^2^ and ANOVA, *p*-value		5.19, 0.023		3.06, 0.004		1.85, 0.070
**Use of nickname**	12 (48%)	8 (32%)	0.25 (0.46)	0.15 (0.30)	1.05 (1.44)	0.88 (1.54)
*χ*^2^ and ANOVA, *p*-value		1.33, 0.248		0.83, 0.410		0.41, 0.687
**Use of real name**	16 (64%)	15 (60%)	1.30 (2.01)	0.43 (0.48)	1.57 (1.52)	1.69 (1.98)
*χ*^2^ and ANOVA, *p*-value		0.08, 0.771		2.09, 0.042		−0.24, 0.813
**Reference to self**	18 (72%)	18 (72%)	0.56 (0.61)	0.36 (0.51)	1.96 (1.36)	1.65 (1.50)
*χ*^2^ and ANOVA, *p*-value		–		1.22, 0.229		0.77, 0.444
**Global speech** ^*^	<5 (–)^*^	<5 (–)^*^	0.01 (0.04)	0.04 (0.10)	0.12 (0.58)	0.20 (0.63)
*χ*^2^ and ANOVA, *p*-value		2.00, 0.157		−1.50, 0.139		−0.49, 0.629
**Specific speech** ^*^	<5 (–)^*^	9 (36%)	0.04 (0.13)	0.13 (0.23)	0.54 (1.76)	0.67 (1.28)
*χ*^2^ and ANOVA, *p*-value		3.95, 0.047		−1.67, 0.101		−0.29, 0.770
**Teaching**	9 (36%)	17 (68%)	0.30 (0.55)	0.33 (0.46)	0.99 (1.50)	2.44 (2.75)
*χ*^2^ and ANOVA, *p*-value		5.13, 0.024		−0.18, 0.854		−2.30, 0.026

EPDS, Edinburgh Postnatal Depression Scale.

^*^ Censored to prevent disclosure because of small cell counts.

^a^ Any vocalisation: any verbal sound made by the infant, including distress, non-distress, screaming, bodily sounds, babbling, first words.

## Results

### Characteristics of the sample

In summary, the majority of children in our sample were first-born, of similar age, with a higher proportion of girls than boys. The average age of mothers and fathers was lower than that reported nationally in the UK [30.7 and 33.6 respectively; (72)] with mothers reporting higher educational attainment compared to fathers. The mean depression score (EPDS) for mothers (Mean = 6.61, SD = 5.37) was similar to that previously reported [Mean = 6.34, SD = 4.33; (73)], while the mean depression score for fathers in our sample (Mean = 8.17, SD = 5.11) was higher in comparison with previous research [Mean = 4.35, SD = 3.72; (73)] (Table 2).

### Descriptive characteristics of infant and parental vocalisation behaviours

Frequency, mean RPM, and mean duration of parental and infant vocalisation behaviours are displayed in Table 3. Overall, there were no differences in frequency and duration of infant vocalisation behaviours during father-infant, compared to mother-infant, interactions. Non-distress and any vocalisation (any verbal sound made by the infant, including distress, non-distress, screaming, bodily sounds, babbling and first words) were the most frequent behaviours displayed by the infants. Similarly, there were no differences between frequency and duration of maternal and paternal vocalisation behaviours when interacting with their infants. The most frequently demonstrated maternal and paternal vocalisations were speech, non-verbal sounds, silence and laughter, which also had the longest duration. With regard to modifiers of parental speech, the most frequent sentence structures were statements and questions, with both mothers and fathers acknowledging their infants during interactions. Fathers demonstrated higher frequency and mean RPM of commands and exclamations compared to mothers. Both mothers and fathers used positive and neutral tone, with somewhat higher mean duration of neutral tone displayed by the mothers compared to fathers. Negative and ironic/sarcastic tone was a rare feature of parental speech, although there was some indication that fathers used ironic/sarcastic tone more frequently than mothers. Both mothers and fathers engaged in adult- and infant-directed speech, although mean duration of adult-directed speech was higher, and mean duration of infant directed speech was lower, in fathers compared to mothers. Both mothers and fathers praised their infants during interactions, however, the frequency, mean RPM and duration of criticism directed toward the infant was higher in fathers than mothers. Similarly, frequency and mean duration of intrusive vocalisation behaviours was higher in fathers compared to mothers. Despite higher frequency and duration of criticism and intrusiveness, fathers engaged in attuned mind-mindedness more often, and for longer duration, compared to mothers. High frequency of infant-directed encouragement and low frequency of infant discouragement was displayed by both mothers and fathers. Frequency and mean RPM of verbal play was higher in fathers than mothers. Both mothers and fathers addressed their infants by their real name more often than the nickname, with some indications that fathers had higher mean RPM of using infant's real name compared to mothers. Both frequency and mean duration of teaching was higher in mothers compared to fathers. There was no evidence to suggest that paternal and maternal PND were correlated with the frequency of infant vocalisation behaviours, including distress, non-distress or any infant vocalisation (Table 4).

**Table 4:** Correlations between paternal and maternal PND and infant vocalisations.

Infant vocalisation	Correlations with paternal PND (EPDS; *n* = 23)	Correlations with maternal PND (EPDS; *n* = 23)
Frequency of infant distress	*r* = 0.08 (−0.45, 0.62), *p* = 0.752	*r* = −0.14 (−0.51, 0.21), *p* = 0.49
Frequency of infant non-distress	*r* = 0.31 (−0.07, 0.70), *p* = 0.111	*r* = −0.33 (−0.64, 0.08), *p* = 0.10
Frequency of any infant vocalisation^a^	*r* = 0.06 (−0.38, 0.51), *p* = 0.773	*r* = −0.09 (−0.47, 0.32), *p* = 0.66

EPDS, Edinburgh Postnatal Depression Scale; PND, postnatal depression.

^a^ Any infant vocalisation: any verbal sound made by the infant, including distress, non-distress, screaming, bodily sounds, babbling, first words.

### Associations between parental PND and vocalisation behaviours

There was no evidence for associations between maternal PND and the majority of vocalisations in hypothesis-led analyses. There was evidence for a reduction in the frequency of maternal neutral tone as maternal PND increased in the unadjusted and adjusted models (*β*: −0.11, 95% CI: −0.20, −0.02, *p* = 0.016; Tables 5, 6). There was also evidence that increases in maternal PND were associated with increased odds of being in a quantile with higher duration of positive tone in models adjusting for amount of speech directed to the infant and duration of infant vocalisation (POR: 1.20, 95% CI: 1.02, 1.40, *p* = 0.026), and fully adjusted models (POR: 1.28, 95% CI: 1.04, 1.56, *p* = 0.017). There were fewer than five mothers displaying negative tone, precluding from examining associations with PND using inferential statistics.

**Table 5:** Linear regressions of hypothesis-led analysis of associations between maternal and paternal PND and parental vocalisation behaviours.

Parental vocalisation behaviours	Paternal PND (EPDS; *n* = 23)			
	Unadjusted^a^	Adjusted for amount of speech directed to infant^b^	Further adjusted for frequency or mean of infant vocalisation^c^	Further adjusted for maternal, paternal and child characteristics^d^
	B [95% CI], *p*-value	B [95% CI], *p*-value	B [95% CI], *p*-value	B [95% CI], *p*-value
Frequency of paternal speech	−0.01 [−0.28, 0.27], *p* = 0.966	−0.02 [−0.22, 0.17], *p* = 0.800	−0.02 [−0.22, 0.18], *p* = 0.839	0.02 [−0.27, 0.32], *p* = 0.878
Frequency of paternal neutral tone	0.01 [−0.03, 0.04], *p* = 0.773	0.01 [−0.03, 0.04], *p* = 0.781	0.01 [−0.03, 0.04], *p* = 0.795	−0.01 [−0.06, 0.04], *p* = 0.567
Duration of paternal neutral tone	0.03 [−0.03, 0.09], *p* = 0.320	0.03 [−0.03, 0.09], *p* = 0.323	0.02 [−0.03, 0.08], *p* = 0.363	0.03 [−0.06, 0.11], *p* = 0.520
	Maternal PND (EPDS; *n* = 23)			
Frequency of maternal speech	−0.13 [−0.49, 0.13], *p* = 0.309	−0.11 [−0.34, 0.12], *p* = 0.335	−0.10 [−0.33, 0.13], *p* = 0.380	−0.09 [−0.36, 0.17], *p* = 0.471
Frequency of maternal neutral tone	−0.11 [−0.20, −0.02], *p* = 0.016	−0.11 [−0.20, −0.03], *p* = 0.010	−0.12 [−0.20, −0.03], *p* = 0.011	−0.10 [−0.19, −0.002], *p* = 0.045

EPDS, Edinburgh Postnatal Depression Scale; PND, postnatal depression.

^a^ Unadjusted model containing exposure and outcome only.

^b^ Adjusted for amount of caregiver speech directed to infant.

^c^ Further adjusted for frequency or mean duration of infant vocalisation.

^d^ Further adjusted for maternal and paternal (age at birth and education) and child (sex) characteristics.

**Table 6:** Ordered logistic regression of hypothesis-led analysis of associations between maternal and paternal depressive symptoms and caregiver vocalisations.

Caregiver vocalisation	Paternal PND (EPDS; *n* = 23)			
	Unadjusted^a^	Adjusted for amount of speech directed to infant^b^	Further adjusted for frequency or mean of infant vocalisation^c^	Further adjusted for maternal, paternal and child characteristics^d^
	POR [95% CI], *p*-value	POR [95% CI], *p*-value	POR [95% CI], *p*-value	POR [95% CI], *p*-value
Duration of paternal speech	1.07 [0.93, 1.23], *p* = 0.362	1.06 [0.92, 1.23], *p* = 0.380	1.07 [0.93, 1.23], *p* = 0.369	1.28 [1.01, 1.63], *p* = 0.045
Frequency of paternal infant register	1.01 [0.88, 1.16], *p* = 0.881	1.01 [0.88, 1.16], *p* = 0.867	1.02 [0.88, 1.18], *p* = 0.816	1.01 [0.80, 1.27], *p* = 0.923
Duration of paternal infant register	1.12 [0.97, 1.29], *p* = 0.131	1.12 [0.97, 1.29], *p* = 0.120	1.13 [0.97, 1.30], *p* = 0.104	1.27 [1.00, 1.61], *p* = 0.046
Frequency of paternal mind-mindedness	1.07 [0.94, 1.25], *p* = 0.353	1.08 [0.92, 1.25], *p* = 0.349	1.07 [0.92, 1.25], *p* = 0.395	0.93 [0.74, 1.17], *p* = 0.556
Duration of paternal mind-mindedness	0.99 [0.86, 1.14], *p* = 0.872	0.98 [0.85, 1.13], *p* = 0.793	0.98 [0.84, 1.13], *p* = 0.764	0.86 [0.68, 1.08], *p* = 0.202
Frequency of paternal positive tone	1.20 [1.02, 1.41], *p* = 0.032	1.20 [1.02, 1.42], *p* = 0.031	1.24 [1.04, 1.47], *p* = 0.014	1.28 [1.02, 1.61], *p* = 0.037
Duration of paternal positive tone	1.09 [0.94, 1.27], *p* = 0.230	1.14 [0.97, 1.33], *p* = 0.108	1.17 [0.98, 1.38], *p* = 0.069	1.21 [0.94, 1.55], *p* = 0.129
	Maternal PND (EPDS; *n* = 23)			
Duration of maternal speech	1.01 [0.88, −0.8], *p* = 0.934	1.01 [0.88, 1.15], *p* = 0.933	1.03 [0.90, 1.19], *p* = 0.639	1.10 [0.93, 1.30], *p* = 0.246
Frequency of maternal infant register	0.93 [0.82, 1.06], *p* = 0.290	0.90 [0.78, 1.04], *p* = 0.170	0.90 [0.77, 1.04], *p* = 0.138	0.90 [0.77, 1.05], *p* = 0.173
Duration of maternal infant register	1.01 [0.89, 1.15], *p* = 0.873	1.01 [0.89, 1.16], *p* = 0.839	1.03 [0.89, 1.18], *p* = 0.718	1.04 [0.89, 1.21], *p* = 0.647
Frequency of maternal mind-mindedness	1.10 [0.97, 1.26], *p* = 1.141	1.10 [0.97, 1.25], *p* = 0.147	1.09 [0.96, 1.25], *p* = 0.187	1.08 [0.95, 1.24], *p* = 0.243
Duration of maternal mind-mindedness	1.10 [0.96, 1.26], *p* = 0.158	1.10 [0.96, 1.26], *p* = 0.160	1.14 [0.98, 1.32], *p* = 0.083	1.12 [0.96, 1.31], *p* = 0.155
Frequency of maternal positive tone	0.92 [0.81, −0.57], *p* = 0.237	0.93 [0.81, 1.07], *p* = 0.294	0.93 [0.81, 1.07], *p* = 0.295	0.94 [0.80, 1.09], *p* = 0.361
Duration of maternal positive tone	1.13 [0.98, 1.29], *p* = 0.092	1.14 [0.98, 1.31], *p* = 0.082	1.20 [1.02, 1.40], *p* = 0.026	1.28 [1.04, 1.56], *p* = 0.017
Duration of maternal neutral tone	1.01 [0.88, 1.16], *p* = 0.845	1.04 [0.90, 1.21], *p* = 0.590	1.08 [0.92, 1.26], *p* = 0.328	1.10 [0.91, 1.32], *p* = 0.313

POR, proportional odds ratio; EPDS, Edinburgh Postnatal Depression Scale; PND, postnatal depression.

^a^ Unadjusted model containing exposure and outcome only.

^b^ Adjusted for amount of caregiver speech directed to infant.

^c^ Further adjusted for frequency or mean duration of infant vocalisation.

^d^ Further adjusted for maternal and paternal (age at birth and education) and child (sex) characteristics.

Within hypothesis-free analyses of maternal vocalisations, there was only evidence for an association between maternal PND and duration of maternal encouragement. After adjusting for amount of speech directed to the infant, there was evidence that increased maternal PND was associated with increased odds of being in a quantile that encouraged their children for longer periods of time (POR: 0.08, 95% CI: 0.01, 0.15, *p* = 0.036). There was also weak evidence for this association when adjusting for amount of speech directed at the infant and mean duration of infant vocalisation (POR: 0.07, 95% CI: −0.003, 0.15, *p* = 0.06).

Similarly to mothers, there was no evidence of associations between paternal PND and the majority of paternal vocalisation behaviours in hypotheses-led analyses (Tables 5, 6). However, there was some evidence that paternal PND was associated with higher odds of being in a quantile with higher frequency of paternal positive tone in the unadjusted and fully adjusted ordered logistic regression models [Proportional Odds Ratio (POR): 1.28, 95% CI: 1.02, 1.61, *p* = 0.037; Table 6], although confidence intervals were wide. Paternal PND was also associated with higher odds of being in a quantile with higher mean duration of paternal speech (POR: 1.28, 95% CI: 1.01, 1.63, *p* = 0.045; Table 6) and infant-directed speech (POR: 1.27, 95% CI: 1.00, 1.61, *p* = 0.046; Table 6), however this was only apparent in fully adjusted ordered logistic regressions models accounting for maternal, paternal and child characteristics.

Hypotheses-free analyses of modifiers of paternal speech revealed no evidence for associations between paternal PND and the majority of paternal speech modifiers (Table 7). However, paternal PND was associated with higher odds of being in a quantile with higher frequency (POR: 1.52, 95% CI: 1.13, 2.05, *p* = 0.005; Table 7) and mean duration (POR: 1.63, 95% CI: 1.18, 2.25, *p* = 0.003; Table 7) of paternal laugh in the unadjusted and fully adjusted ordered logistic regression models. Paternal PND was also associated with higher odds of being in a quantile with higher mean duration of paternal question (POR: 1.79, 95% CI: 1.19, 2.70, *p* = 0.005; Table 7) and encouragement (POR: 1.25, 95% CI: 0.98, 1.60, *p* = 0.073; Table 7), however, this was only evident in fully adjusted ordered logistic regression models accounting for maternal, paternal and child characteristics.

**Table 7:** Ordered logistic regressions hypothesis-free analyses of modifiers^*^ of paternal and maternal speech.

Modifiers of caregiver speech	Paternal PND (EPDS; *n* = 23)			
	Unadjusted^a^	Adjusted for amount of speech directed to infant^b^	Further adjusted for frequency or mean of infant vocalisation^c^	Further adjusted for maternal, paternal and child characteristics^d^
	POR [95% CI], *p*-value	POR [95% CI], *p*-value	POR [95% CI], *p*-value	POR [95% CI], *p*-value
Frequency of paternal laugh	1.17 [1.00, 1.37], *p* = 0.058	1.17 [1.00, 1.36], *p* = 0.050	1.21 [1.02, 1.44], *p* = 0.027	1.52 [1.13, 2.05], *p* = 0.005
Duration of paternal laugh	1.20 [1.03, 1.41], *p* = 0.022	1.20 [1.03, 1.41], *p* = 0.021	1.21 [1.03, 1.42], *p* = 0.021	1.63 [1.18, 2.25], *p* = 0.003
Frequency of paternal command	0.99 [0.86, 1.14], *p* = 0.883	0.97 [0.83, 1.12], *p* = 0.661	0.97 [0.83, 1.12], *p* = 0.670	0.96 [0.75, 1.23], *p* = 0.775
Duration of paternal command	1.06 [0.91, 1.23], *p* = 0.447	1.06 [0.91, 1.23], *p* = 0.446	1.06 [0.91, 1.23], *p* = 0.435	1.23 [0.95, 1.59], *p* = 0.112
Frequency of paternal question	0.96 [0.84, 1.10], *p* = 0.603	0.96 [0.84, 1.11], *p* = 0.613	0.96 [0.84, 1.11], *p* = 0.614	0.91 [0.74, 1.13], *p* = 0.398
Duration of paternal question	1.12 [0.96, 1.29], *p* = 0.139	1.12 [0.96, 1.30], *p* = 0.132	1.12 [0.96, 1.30], *p* = 0.146	1.79 [1.19, 2.70], *p* = 0.005
Frequency of paternal exclamation	1.03 [0.89, 1.19], *p* = 0.670	1.03 [0.89, 1.19], *p* = 0.666	1.03 [0.89, 1.19], *p* = 0.654	1.17 [0.93, 1.48], *p* = 0.182
Duration of paternal exclamation	1.00 [0.86, 1.13], *p* = 0.866	1.00 [0.86, 1.13], *p* = 0.844	1.00 [0.86, 1.13], *p* = 0.890	1.21 [0.95, 1.54], *p* = 0.124
Frequency of paternal statement	0.97 [0.84, 1.12], *p* = 0.705	0.95 [0.82, 1.11], *p* = 0.556	0.95 [0.81, 1.11], *p* = 0.539	0.96 [0.77, 1.19], *p* = 0.684
Duration of paternal statement	1.03 [0.88, 1.22], *p* = 0.666	1.04 [0.88, 1.22], *p* = 0.671	1.02 [0.87, 1.21], *p* = 0.761	1.15 [0.90, 1.46], *p* = 0.276
Frequency of paternal praise of infant	1.11 [0.95, 1.30], *p* = 0.194	1.09 [0.93, 1.28], *p* = 0.279	1.10 [0.93, 1.29], *p* = 0.254	1.12 [0.88, 1.42], *p* = 0.370
Duration of paternal praise of infant	1.20 [1.03, 1.41], *p* = 0.020	1.20 [1.03, 1.40], *p* = 0.023	1.20 [1.03, 1.41], *p* = 0.022	1.23 [0.98, 1.55], *p* = 0.076
Frequency of paternal encouragement	0.95 [0.83, 1.10], *p* = 0.519	0.93 [0.80, 1.08], *p* = 0.356	0.93 [0.80, 1.08], *p* = 0.348	0.82 [0.65, 1.05], *p* = 0.113
Duration of paternal encouragement	1.10 [0.95, 1.28], *p* = 0.196	1.10 [0.95, 1.28], *p* = 0.209	1.11 [0.95, 1.30], *p* = 0.174	1.25 [0.98, 1.60], *p* = 0.073
	Maternal PND (EPDS; *n* = 23)			
Frequency of maternal laugh	0.98 [0.86, 1.12], *p* = 0.747	1.10 [0.87, 1.14], *p* = 0.993	1.10 [0.87, 1.14], *p* = 0.955	1.00 [0.86, 1.18], *p* = 0.954
Duration of maternal laugh	1.02 [0.89, 1.16], *p* = 0.816	1.02 [0.89, 1.18], *p* = 0.727	1.05 [0.91, 1.21], *p* = 0.483	1.07 [0.91, 1.26], *p* = 0.425
Frequency of maternal command	0.88 [0.76, 1.02], *p* = 0.092	0.84 [0.71, 1.00], *p* = 0.057	0.84 [0.71, 1.00], *p* = 0.057	0.86 [0.70, 1.05], *p* = 0.131
Duration of maternal command	0.91 [0.79, 1.05], *p* = 0.198	0.91 [0.78, 1.05], *p* = 0.187	0.89 [0.77, 1.04], *p* = 0.159	0.90 [0.76, 1.06], *p* = 0.198
Duration of maternal question	1.06 [0.91, 1.22], *p* = 0.454	1.06 [0.91, 1.22], *p* = 0.453	1.05 [0.91, 1.21], *p* = 0.512	1.14 [0.96, 1.36], *p* = 0.122
Frequency of maternal exclamation	0.94 [0.81, 1.10], *p* = 0.458	0.95 [0.81, 1.12], *p* = 0.567	0.95 [0.80, 1.13], *p* = 0.562	1.05 [0.86, 1.28], *p* = 0.606
Duration of maternal exclamation	0.93 [0.80, 1.08], *p* = 0.351	0.90 [0.76, 1.07], *p* = 0.226	0.90 [0.76, 1.08], *p* = 0.269	0.99 [0.80, 1.22], *p* = 0.916
Duration of maternal statement	1.05 [0.92, 1.20], *p* = 0.497	1.05 [0.92, 1.21], *p* = 0.447	1.09 [0.94, 1.26], *p* = 0.266	1.26 [1.02, 1.56], *p* = 0.032
Frequency of maternal praise of infant	0.96 [0.84, 1.10], *p* = 0.589	1.06 [0.88, 1.28], *p* = 0.550	1.08 [0.88, 1.32], *p* = 0.454	0.97 [0.75, 1.26], *p* = 0.823
Duration of maternal praise of infant	1.00 [0.88, 1.13], *p* = 0.959	1.02 [0.89, 1.18], *p* = 0.737	1.02 [0.89, 1.18], *p* = 0.747	0.93 [0.77, 1.12], *p* = 0.428
Frequency of maternal encouragement	0.93 [0.81, 1.07], *p* = 0.310	0.93 [0.81, 1.08], *p* = 0.356	0.94 [0.81, 1.09], *p* = 0.403	0.94 [0.80, 1.10], *p* = 0.428

POR, proportional odds ratio; EPDS, Edinburgh Postnatal Depression Scale; PND, postnatal depression.

^a^ Unadjusted model containing exposure and outcome only.

^b^ Adjusted for amount of caregiver speech directed to infant.

^c^ Further adjusted for frequency or mean duration of infant vocalisation.

^d^ Further adjusted for maternal and paternal (age at birth and education) and child (sex) characteristics.

^*^ Modifiers allow for more detailed categorisation within behavioural group, in this instance maternal and paternal speech.

There was no evidence to suggest that paternal PND was associated with any of the rare modifiers of paternal speech, including paternal discouragement and criticism of the infant, negative and ironic/sarcastic tone, intrusiveness, use of humour and teaching the infant (Table 8).

**Table 8:** Logistic regressions analyses of rare modifiers^*^ of paternal speech (dichotomised; behaviour did vs. did not occur).

Modifiers of caregiver speech	Paternal PND (EPDS; *n* = 23)			
	Unadjusted^a^	Adjusted for amount of speech directed to infant^b^	Further adjusted for frequency or mean of infant vocalisation^c^	Further adjusted for maternal, paternal and child characteristics^d^
	OR [95% CI], *p*-value	OR [95% CI], *p*-value	OR [95% CI], *p*-value	OR [95% CI], *p*-value
Paternal infant discouragement	0.96 [0.81, 1.13], *p* = 0.613	0.95 [0.80, 1.13], *p* = 0.572	0.95 [0.80, 1.13], *p* = 0.589	0.84 [0.62, 1.14], *p* = 0.265
Paternal criticism of infant	1.03 [0.87, 1.21], *p* = 0.721	1.03 [0.87, 1.22], *p* = 0.742	1.03 [0.87, 1.22], *p* = 0.730	0.92 [0.71, 1.20], *p* = 0.554
Paternal negative tone	1.03 [0.85, 1.25], *p* = 0.752	1.03 [0.85, 1.25], *p* = 0.742	1.05 [0.85, 1.29], *p* = 0.650	1.07 [0.75, 1.52], *p* = 0.706
Paternal intrusiveness	1.01 [0.85, 1.19], *p* = 0.973	1.00 [0.85, 1.19], *p* = 0.973	1.00 [0.84, 1.19], *p* = 0.985	1.01 [0.70, 1.45], *p* = 0.966
Paternal sarcasm	1.00 [0.82, 1.17], *p* = 0.834	1.00 [0.83, 1.17], *p* = 0.869	1.00 [0.83, 1.18], *p* = 0.917	1.01 [0.77, 1.33], *p* = 0.926
Paternal use of humour	1.03 [0.86, 1.23], *p* = 0.711	1.04 [0.85, 1.27], *p* = 0.714	1.10 [0.82, 1.48], *p* = 0.521	0.90 [0.59, 1.36], *p* = 0.620
Paternal teaching	1.19 [1.00, 1.48], *p* = 0.109	1.19 [1.00, 1.48], *p* = 0.115	1.19 [1.00, 1.49], *p* = 0.114	1.10 [0.76, 1.60], *p* = 0.600

OR, odds ratio; EPDS, Edinburgh Postnatal Depression Scale; PND, postnatal depression.

^a^ Unadjusted model containing exposure and outcome only.

^b^ Adjusted for amount of caregiver speech directed to infant.

^c^ Further adjusted for frequency or mean duration of infant vocalisation.

^d^ Further adjusted for maternal and paternal (age at birth and education) and child (sex) characteristics.

^*^ Modifiers allow for more detailed categorisation within behavioural group, in this instance maternal and paternal speech.

## Discussion

### Main findings

In line with previous research, we found that there were more similarities than differences between maternal and paternal vocalisation behaviours (45). Fathers and mothers both engage in equal frequency and duration of vocalisation behaviours when interacting with their infants, with speech, non-verbal sounds, silence and laughter being the most frequent vocalisations. There were also similarities in sentence structure, such as use of statements and questions, with both mothers and fathers acknowledging their infant during interactions. Both mothers and fathers used positive and neutral tone, although the mean duration of maternal neutral tone was higher than that for fathers. High frequency of encouragement of infant behaviours and low frequency of infant discouragement was observed in both mothers and fathers, with both addressing their infants by their real name more frequently than a nickname.

There were also some notable differences between maternal and paternal vocalisation behaviours. For instance, fathers demonstrated higher frequency and mean duration of commands and exclamation compared to mothers, while mothers engaged in more teaching compared to fathers. Existing research on content and function of maternal and paternal speech has documented a similar pattern, with fathers producing more direct (74) and prohibition commands than mothers (75). In line with previous research, fathers in our study engaged in more verbal play (both frequency and mean duration) compared to mothers (76). Adult- and infant-directed speech was a feature of both maternal and paternal vocalisation behaviours, though the mean duration of adult-directed speech was higher and that of infant-directed speech was lower in fathers compared to mothers. Existing research on paternal infant-directed speech to preverbal infants is rare. There is some evidence to suggest that, similarly to mothers, fathers make some prosodic modifications, but to a lesser extent. This is possibly due to men's lower average pitch compared to women's (45, 77).

Even though negative and ironic/sarcastic tone was a rare feature of parental speech, there was some evidence that this was more frequently displayed by fathers than mothers. This finding is consistent with earlier studies suggesting that fathers are more likely to tease their children compared to mothers (78). Similarly, frequency and duration of both criticism of the child and verbal intrusiveness was higher in fathers compared to mothers. Given the exploratory nature of our study, these findings should be interpreted with caution. However, these findings are in line with existing research suggesting that fathers may be less sensitive, more intrusive, directive and parent-centred than mothers during parent-infant interactions (79). It is possible that fathers engage in a more direct, stimulating, and challenging style of father-child interactions, so called “activation” parenting (80), which may seem more intrusive, but is important for promoting social and cognitive competencies in children (117). It has been previously argued that moderate levels of intrusiveness combined with positive and challenging stimulation in a supportive context that characterise fathers' interactional style allows children to explore new horizons while feeling safe and protected (82–84). It has also been argued that maternal and paternal sensitivity may be expressed differently through emotional warmth (mothers) and physical stimulation and playful interactions [fathers; (85)]. It has been argued that these two types of experiences chart the “rhythm of safety” vs. the “rhythm of exploration”, both of which are essential for healthy infant development (86). Future research that examines sequences of parental and child behaviours will enable us to examine behavioural manifestations of paternal intrusiveness in combination with other interaction behaviours (e.g., positive and/or negative affect) and, subsequently, their potentially differential effect on child development (87). This may lead to a reframing in how intrusive behaviour is defined and applied in the context of father-child interactions, and what it means for child development.

Despite higher frequency and duration of criticism and intrusiveness, fathers in our study engaged in mind-mindedness more often and for longer duration than mothers. Existing research on mind-minded speech in mothers and fathers is somewhat inconsistent, with some studies reporting no differences in the overall frequency of mind-minded comments between mothers and fathers (88), while other earlier studies noting more attentive utterances produced by fathers compared to mothers (89).

Based on existing literature, we hypothesised that the frequency and duration of maternal and paternal speech, as well as infant-directed and mind-minded speech and positive emotional tone will decrease, while the frequency and duration of negative and neutral emotional tone will increase in the context of parental depression. Our findings did not fully support these hypotheses. As hypothesised, there was evidence that as maternal PND increased, the frequency of maternal neutral tone decreased. We found no evidence for an association between paternal PND and use of a neutral tone. However, contrary to our original hypotheses, higher levels of maternal and paternal PND were associated with increased duration of parental positive tone, encouragement and laughter, all of which may be vocal proxies for more positive and sensitive interactions. Similarly, we found evidence to suggest that higher levels of paternal PND were also associated with increased duration of paternal speech, as well as infant-directed speech and questions, which may be suggestive of more infant-centred interactions.

Several explanations may be put forward to contextualise our findings. Existing research suggests that fathers with clinical levels of depression may engage in less positive parenting practices, and other enriching language activities with the child such as reading, singing songs and telling stories (90). However, effects of paternal PND on parenting, including vocalisation behaviours, may be more subtle in community samples with relatively mild levels of depression (91), and may not necessarily present themselves as overtly negative vocalisations (overall rare in our sample). Both neutral and positive tone were a predominant feature of maternal and paternal speech, whilst negative tone was rare with only 4% of fathers and 5% of mothers using a negative tone. It may be that there was more variability in neutral and positive tone vocalisations to detect effects associated with parental PND, with the opposite true for the negative parental tone. It is also possible that those parents who experience mild levels of PND have more emotional insight and empathy, and, thus, engage in more sensitive parenting, including vocalisation behaviours. It is possible that parents with increased PND try to compensate for their low mood by speaking in a positive tone, and their tone may not reflect genuine positive emotion. It is possible that more global qualitative coding such as the Emotional Availability Scales which capture an overall emotional tone would find differential results and this would be an interesting future direction to test this hypothesis. In addition, the focus of our analyses was the associations between parental PND and the frequency and duration of parental vocalisation behaviours, but sequences and patterns of behaviours may better capture patterns of expressed depressed mood.

Another contextual framework to explain our findings may be the changing nature of beliefs and attitudes surrounding contemporary parenting practices. Sociological literature has meticulously documented changes in parenting that occurred in the last forty years (92). The phenomenon of intensive or hyper-parenting (93), a highly demanding and child-centred approach to parenting, which includes heightened parental awareness of the adverse consequences of “bad parenting” ranging from poor educational to developmental outcomes (93, 94). Consequently, the process of raising a child through “concerted cultivation” (95) and attentive parenting may offer a way of mitigating the risks (94), including those associated with adverse parental mental health [for the critique and contested nature of such parenting practices see (96–99)]. Although the term “parenting” may be gender-neutral, the lens of intensive parenting has been predominantly focused on mothers as the primary agents responsible for shaping child outcomes (98, 100). However, the expectation that fathers should also be involved in parenting and childcare has also become prevailing in contemporary society (101, 102), with the demands of intensive parenting, albeit not to the same degree as mothers (101, 103), also extending to men. Intensive parenting is embedded in middle-class values (104), with “concerted cultivation” made possible through higher levels of financial and educational resources, with higher parental socio-economic status being consistently associated with more positive and consistent parenting styles and practices (105). Based on this evidence, it may be possible that both mothers and fathers in our study modified their vocalisation behaviours in line with their knowledge of the effects of mental health on parenting and the child, or there were more likely to engage in attentive and child-centred parenting in line with contemporary assumptions on what it means to be a “good” parent. Future longitudinal research with larger samples is needed to corroborate our findings regarding parental depression and vocalisation behaviours, particularly in the context of beliefs and expectations surrounding contemporary parenting practices.

### Strengths and limitations

One of the strengths of the study is the assessment of both maternal and paternal PND and vocalisation behaviours in early infancy. Most of the research to date has focused on maternal PND and its impact on parenting (2), with only a few studies addressing the impact of paternal PND on vocalisation behaviours during father-infant interactions. This approach continues to perpetuate a potentially problematic stance in developmental psychology, placing the mother-infant relationship at the cornerstone of human development. Similarly to mothers, features of father-infant vocalisation interactions may also constitute a transmission pathway by which paternal PND impacts the child. This argument is in line with the recently articulated stance to consider both maternal and paternal mental health from a family system perspective for both research and intervention purposes (106). Our comparisons of maternal and paternal vocalisation behaviours suggest that there are more similarities than differences between maternal and paternal vocal interactions with the infant, with comparative effects of parental PND on some aspects of vocalisation behaviours.

The use of observational rather than parent-reported measures to assess parental vocalisation behaviours is another strength of this study. The association between parental PND and the reporting of parenting has been consistently supported (107). In addition, there is some evidence to suggest that the headcams may be better at capturing vocalisation behaviours compared to “gold standard” observational methods due to the build-in microphones positioned closer to the participants' face (52). Importantly, the use of headcams is more likely to reduce participant reactivity and demand characteristics, enabling us to capture less socially desirable behaviours, including parental vocalisations (52). Although the advantage of reducing demand characteristics is not specific to the headcams, recording interactions in the familiar setting of a home context without a researcher present may facilitate capturing more variability in parental behaviours, compared to traditional observational methods. The use of the headcams may also reduce parental social anxiety and feelings of being judged, particularly in those parents who experience depression, compared to “gold standard” observational methods with a researcher filming the interactions, facilitating more natural positive responses to the child.

We captured a wide range of parental vocalisation behaviours, including a more detailed categorisation of each vocalisation withing a wider behavioural group using event-based micro-coding scheme (58). Arguably, the micro-coding systems may be better at highlighting complex patterns of dyadic interactions, capturing behaviours from both parents and infants as active participants in the interaction (108). It has also been argued that in comparison to global ratings, the systematic nature of micro-behavioural coding allows for the capture of more precise information on the nature of the observed behaviours (109). Indeed, our micro-coding behavioural scheme enabled us to capture multiple dimensions of parental and infant vocalisation with unprecedented degree of granularity regarding parental vocalisation behaviours (110, 111). Given the lack of evidence regarding the impact of maternal and, particularly, paternal depression on their vocalisation behaviours, such a degree of detail is particularly important.

The detailed nature of the micro-coding scheme has also enabled us to build a comprehensive comparison of maternal and paternal vocalisation behaviours across a range of dimensions, including their variation in intensity and frequency. Arguably, the use of headcams during mealtime and play interactions as they naturally take place at home, combined a multidimensional and versatile micro-coding behavioural scheme may be a better methodological paradigm to capture and assess the dynamic and transactional nature of parent-child interactions.

It should be noted, however, that our study is explorative in nature and findings should be interpreted with caution due to the fact that the study was not adequately powered to detect the observed effect sizes – the relatively small sample size and multiple testing with parental vocalisations as outcomes increased the possibility of committing a type 1 error. In addition, vocalisation behaviours made by fewer than five mothers and fathers had to be removed from the analyses, thus, associations between maternal and paternal PND and such behaviours could not be investigated. Further replication studies with a larger sample are needed to substantiate our preliminary findings, as well as provide more insights into the effects of parental PND on more rare aspects of parental vocalisations (e.g., maternal criticism of infant and intrusiveness). In addition, there were some discrepancies between the nature of the tasks completed between mothers and fathers, with mothers predominantly engaging in mealtime and stacking tasks, whilst a proportion of fathers have also engaged in a free play task. This could potentially elicit different type of parental vocalisation behaviours, with more goal-oriented interactions (e.g., mealtime and stacking task) eliciting vocalisations that are not necessarily generalisable across all parent-child interactions. Fathers engaging in free play task may also elicit parental vocalisations that are not directly comparable to less physically arousing and task-oriented interactions that mothers and infants accomplished. However, recent comparisons of studies between maternal and paternal vocalisation behaviours suggest that, despite some differences, both mothers and fathers modify their speech depending on the nature of the interaction (45).

Another limitation of the study relates to potential selection bias. The ALSPAC cohort is now a three-generational study, comprising “G0”: the cohort of original pregnant women, the biological father and other carers/partners, “G1”: the cohort of index children, and “G2”: the cohort of offspring of the index children, from which our study sample was drawn. The G0 mothers are overall from somewhat higher socio-economic background compared to the general population, whilst G1 participants who enrolled their children in G2 are more engaged with the ALSPAC study and more educated compared to those who did not participate in the study (55). By design, G1 participants fall within a restricted age range, with maternal age being further restricted by missing very young mothers who were not recruited, and the average age of mothers and fathers in this sample was lower than that reported nationally in the UK [30.7 and 33.6 respectively; (72)]. Both maternal age and education have been previously found to be important confounders in the association between parental depression, offspring outcomes and parenting (68, 63). It may be possible that infants of older and more educated parents are less likely to be exposed to specific manifestations of reduced parental insensitivity, including more negative vocalisation behaviours, associated with parental depression (112, 113). Fathers in our sample had higher mean depression scores than those previously reported, with men scoring on average 2 points above women. Anecdotally, it may be possible that fathers who experienced mental health difficulties were more likely to engage with the headcams study because of the insights it may bring into their parenting and potential impact on the child.

Associations among parental mental health, parenting and offspring development are complex and bidirectional (114). In line with transactional developmental models (115), children with more difficult temperament may influence maternal and paternal PND, as well as parental behavioural responses (116). We attempted to account for possible evocative effects by adjusting our analyses for the frequency and duration of infant vocalisation. However, addressing the possible bidirectionality (117) was outside the scope of the present study.

### Conclusions, implications, and future research

Pathways between maternal and paternal PND, parenting behaviours and offspring outcomes are complex and not fully elucidated. Our findings relate to one aspect of these complex relationships, notably the impact of parental PND on specific manifestations of vocalisation behaviours. Descriptively, we found more similarities than differences between maternal and paternal vocal interactions with their infants, with comparative effects of parental PND on some aspects of vocalisation behaviours, notably positive speech tone, encouragement and laughter. Our findings that higher levels of maternal and paternal PND were associated with increased duration of these behavioural proxies for more positive and sensitive interactions are tentative and implications of the findings are limited. Future replication efforts should focus on larger population-based samples that capture more variability in parental vocalisation behaviours, as well as contemporary beliefs and attitudes that define “good” parenting practices. These findings may indicate that the existing associations between PND and reductions in parental sensitivity require further detailed research.

Future research avenues should also focus on examining associations between specific aspects of parental vocalisation behaviours and more global measures of parental sensitivity to provide further insights into behavioural manifestations of warm and responsive parenting, particularly in the context of parental PND. Better understanding of specific behavioural manifestations of parenting and the overall quality of parent-child relationship and interactions may provide insights into the nature of difficulties that characterise early parent-child interactions, as well as key differences in maternal and paternal behaviours that may indicate depressed mood (61). In addition, the evidence-base regarding the effects of specific parental vocalisation behaviours, as well as patterns and sequences of such vocalisations, on offspring outcomes is lacking and should be strengthened through further investigations.

The coding scheme (58) applied in this study captures an unprecedented range of parental and infant behaviours (e.g., facial expressions, proximity) which should also be addressed in future investigations as potential markers of parental sensitivity which may be affected by parental depression. Traditionally, the main focus in parenting studies has been on mothers, with assessment of paternal parenting based on assessment of maternal parenting and mother-child relationships. Although such strategies may be useful to capture broader aspects of parenting, such as sensitivity and responsiveness, increasingly evidence suggests that the maternal template as a dominant methodology does not capture behaviours that may be unique to fathers, modelling parental sensitivity almost exclusively on maternal behaviours (80). The detailed nature of our coding scheme enables us to build a comprehensive picture of both maternal and paternal behaviours and to capture both differences and similarities in such behaviours across a range of parent-child interactions. It should be noted that even though families may be viewed as organized systems, each individual, including the infant, is an active, contributing member and part of the process that creates and maintains behavioural patterns (117, 118). Thus, the impact of infant temperament and behaviour on parental vocalization behaviours across a range of developmental stages and task scenarios in the context of parental mental health should also be studied.

## Data Availability

The datasets presented in this article are not readily available because ALSPAC data are available through a system of managed open access. The study website contains details of all the data that is available through a fully searchable data dictionary and variable search tool data dictionary. The application steps for ALSPAC data access are highlighted below. (1) Please read the ALSPAC access policy, which describes the process of accessing the data in detail, and outlines the costs associated with doing so. (2) You may also find it useful to browse the fully searchable research proposals database, which lists all research projects that have been approved since April 2011. (3) Please submit your research proposal for consideration by the ALSPAC Executive Committee. You will receive a response within 10 working days to advise you whether your proposal has been approved. If you have any questions about accessing data, please email alspac-data@bristol.ac.uk.

## References

[1] Paulson JF Bazemore SD. Prenatal and postpartum depression in fathers and its association with maternal depression: a meta-analysis. JAMA. (2010) 303(19):1961–9. 10.1001/jama.2010.605 20483973

[2] Stein A Pearson RM Goodman SH Rapa E Rahman A McCallum M Effects of perinatal mental disorders on the fetus and child. Lancet. (2014) 384(9956):1800–19. 10.1016/S0140-6736(14)61277-0 25455250

[3] Bauer A Knapp M Parsonage M. Lifetime costs of perinatal anxiety and depression. J Affect Disord. (2016) 192(1):83–90. 10.1016/j.jad.2015.12.005 26707352

[4] Ramchandani PG Stein A O'Connor TG Heron JON Murray L Evans J. Depression in men in the postnatal period and later child psychopathology: a population cohort study. J Am Acad Child Adolesc Psychiatry. (2008) 47(4):390–8. 10.1097/CHI.0b013e31816429c2 18388761 PMC2650418

[5] Goodman JH. Paternal postpartum depression, its relationship to maternal postpartum depression, and implications for family health. J Adv Nurs. (2004) 45(1):26–35. 10.1046/j.1365-2648.2003.02857.x 14675298

[6] Fredriksen E Von Soest T Smith L Moe V. Depressive symptom contagion in the transition to parenthood: interparental processes and the role of partner-related attachment. J Abnorm Psychol. (2019) 128(5):397–403. 10.1037/abn0000429 30985174

[7] Ainsworth MD Salter MC Blehar EW Sally NW. Patterns of attachment: A psychological study of the strange situation. New York: Psychology Press (2015).

[8] Feldman R Greenbaum CW Mayes LC Erlich SH. Change in mother-infant interactive behavior: relations to change in the mother, the infant, and the social context. Infant Behav Dev. (1997) 20(2):151–63. 10.1016/S0163-6383(97)90018-7

[9] Lovejoy MC Graczyk PA O'Hare E Neuman G. Maternal depression and parenting behavior: a meta-analytic review. Clin Psychol Rev. (2000) 20(5):561–92. 10.1016/S0272-7358(98)00100-7 10860167

[10] Murray L Arteche A Fearon P Halligan S Goodyer I Cooper P. Maternal postnatal depression and the development of depression in offspring up to 16 years of age. J Am Acad Child Adolesc Psychiatry. (2011) 50(5):460–70. 10.1016/j.jaac.2011.02.001 21515195

[11] Wilson S Durbin CE. Effects of paternal depression on fathers’ parenting behaviors: a meta-analytic review. Clin Psychol Rev. (2010) 30(2):167–80. 10.1016/j.cpr.2009.10.007 19926376

[12] Cuijpers P Weitz E Karyotaki E Garber J Andersson G. The effects of psychological treatment of maternal depression on children and parental functioning: a meta-analysis. Eur Child Adolesc Psychiatry. (2015) 24(2):237–45. 10.1007/s00787-014-0660-6 25522839

[13] Galbally M Lewis AJ. Depression and parenting: the need for improved intervention models. Curr Opin Psychol. (2017) 15:61–5. 10.1016/j.copsyc.2017.02.008 28813270

[14] Brookman R Kalashnikova M Conti J Xu Rattanasone N Grant KA Demuth K Depression and anxiety in the postnatal period: an examination of infants’ home language environment, vocalizations, and expressive language abilities. Child Dev. (2020) 91(6):e1211–30. 10.1111/cdev.13421 32745250

[15] Lam-Cassettari C Kohlhoff J. Effect of maternal depression on infant-directed speech to prelinguistic infants: implications for language development. PLoS One. (2020) 15(7):e0236787. 10.1371/journal.pone.0236787 32730322 PMC7392317

[16] Pancsofar N Vernon-Feagans L. Mother and father language input to young children: contributions to later language development. J Appl Dev Psychol. (2006) 27(6):571–87. 10.1016/j.appdev.2006.08.003

[17] Quigley J Nixon E Lawson S. Exploring the association of infant receptive language and pitch variability in fathers’ infant-directed speech. J Child Lang. (2019) 46(4):800–11. 10.1017/S0305000919000175 31023392

[18] Abkarian GG Dworkin JP Abkarian AK. Fathers’ speech to their children: perfect pitch or tin ear? Fathering. (2003) 1(1):27–50. 10.3149/fth.0101.27

[19] Sethna V Murray L Ramchandani PG. Depressed fathers’ speech to their 3-month-old infants: a study of cognitive and mentalizing features in paternal speech. Psychol Med. (2012) 42(11):2361–71. 10.1017/S0033291712000487 22452809

[20] Hart B Risley TR. Meaningful differences in the everyday experience of young American children. Baltimore, MD: Paul H Brookes Publishing (1995).

[21] Murray L Kempton C Woolgar M Hooper R. Depressed mothers’ speech to their infants and its relation to infant gender and cognitive development. J Child Psychol Psychiatry. (1993) 34(7):1083–101. 10.1111/j.1469-7610.1993.tb01775.x 8245134

[22] Allely CS Purves D McConnachie A Marwick H Johnson P Doolin O Parent–infant vocalisations at 12 months predict psychopathology at 7 years. Res Dev Disabil. (2013) 34(3):985–93. 10.1016/j.ridd.2012.11.024 23291516 PMC4046631

[23] Hirsh-Pasek K Adamson LB Bakeman R Owen MT Golinkoff RM Pace A The contribution of early communication quality to low-income children’s language success. Psychol Sci. (2015) 26(7):1071–83. 10.1177/0956797615581493 26048887

[24] Defelipe RP de Resende BD David VF Bussab VSR. Postpartum depression in high-risk Brazilian women: psychosocial predictors and effects on maternal vocalization. Early Child Dev Care. (2019) 189(9):1480–93. 10.1080/03004430.2017.1389918

[25] Reissland N Shepherd J Herrera E. The pitch of maternal voice: a comparison of mothers suffering from depressed mood and non-depressed mothers reading books to their infants. J Child Psychol Psychiatry. (2003) 44(2):255–61. 10.1111/1469-7610.00118 12587861

[26] Treat A Amy E Morris AS Jennifer HG Williamson AC. The impact of positive parenting behaviors and maternal depression on the features of young children's home language environments. J Child Lang. (2020) 47(2):382–400. 10.1017/S030500091900062X 31741432

[27] Santos AK dos Santos LS Bussab VSR. Infant-direct speech and mother-infant attention in depressed and nondepressed mothers. Interação em Psicologia. (2020) 24(1):76–86. 10.5380/psi.v24i1.61959

[28] Spinelli M Fasolo M Mesman J. Does prosody make the difference? A meta-analysis on relations between prosodic aspects of infant-directed speech and infant outcomes. Dev Rev. (2017) 44:1–18. 10.1016/j.dr.2016.12.001

[29] Saint-Georges C Chetouani M Cassel R Apicella F Mahdhaoui A Muratori F Motherese in interaction: at the cross-road of emotion and cognition? (A systematic review). PloS One. (2013) 8(10):e78103. 10.1371/journal.pone.0078103 24205112 PMC3800080

[30] Bettes BA. Maternal depression and motherese: temporal and intonational features. Child Dev. (1988) 59(4):1089–96. 10.2307/1130275 3168616

[31] Porritt LL Zinser MC Bachorowski JA Kaplan PS. Depression diagnoses and fundamental frequency-based acoustic cues in maternal infant-directed speech. Lang Learn Dev. (2014) 10(1):51–67. 10.1080/15475441.2013.802962 24489521 PMC3904677

[32] Kitamura C Lam C. Age-specific preferences for infant-directed affective intent. Infancy. (2009) 14(1):77–100. 10.1080/15250000802569777 32693467

[33] Kaplan PS Bachorowski JA Smoski MJ Hudenko WJ. Infants of depressed mothers, although competent learners, fail to learn in response to their own mothers’ infant directed speech. Psychol Sci. (2002) 13(3):268–71. 10.1111/1467-9280.00449 12009049

[34] Kaplan PS Danko CM Diaz A. A privileged status for male infant-directed speech in infants of depressed mothers? Role of father involvement. Infancy. (2010) 15(2):151–75. 10.1111/j.1532-7078.2009.00010.x 32693473

[35] Scheiber FA Ryckman KK Demir-Lira ÖE. Maternal depressive symptoms and maternal child-directed speech: a systematic review. J Affect Disord. (2022) 297:194–207. 10.1016/j.jad.2021.10.015 34656673 PMC8827171

[36] Meins E. Security of attachment and the social development of cognition. Hove: Lawrence Erlbaum Associates (1997).

[37] Kitamura C Thanavishuth C Burnham D Luksaneeyanawin S. Universality and specificity in infant-directed speech: pitch modifications as a function of infant age and sex in a tonal and non-tonal language. Infant Behav Dev. (2001) 24(4):372–92. 10.1016/S0163-6383(02)00086-3

[38] Meins E Fernyhough C Fradley E Tuckey M. Rethinking maternal sensitivity: mothers’ comments on infants’ mental processes predict security of attachment at 12 months. J Child Psychol Psychiatry Allied Discip. (2001) 42(5):637–48. 10.1017/S0021963001007302 11464968

[39] Pawlby S Fernyhough C Meins E Pariante CM Seneviratne G Bentall RP. Mindmindedness and maternal responsiveness in infant–mother interactions in mothers with severe mental illness. Psychol Med. (2010) 40(11):1861–9. 10.1017/S0033291709992340 20102664

[40] Humphreys KL King LS Choi P Gotlib IH. Maternal depressive symptoms, selffocus, and caregiving behavior. J Affect Disord. (2018) 238:465–71. 10.1016/j.jad.2018.05.072 29929156 PMC6604802

[41] Murray L. The development of children of postnatally depressed mothers: evidence from the Cambridge longitudinal study. Psychoanal Psychother. (2009) 23(3):185–99. 10.1080/02668730903227289

[42] Rebelsky F Hanks C. Fathers’ verbal interaction with infants in the first three months of life. Child Dev. (1971) 42:63–8. 10.2307/1127064 5549515

[43] Kaplan PS Sliter JK Burgess AP. Infant-directed speech produced by fathers with symptoms of depression: effects on infant associative learning in a conditioned-attention paradigm. Infant Behav Dev. (2007) 30(4):535–45. 10.1016/j.infbeh.2007.05.003 17604106 PMC2692315

[44] Tamis-LeMonda CS Shannon JD Cabrera NJ Lamb ME. Fathers and mothers at play with their 2- and 3-year-olds: contributions to language and cognitive development. Child Dev. (2004) 75(6):1806–20. 10.1111/j.1467-8624.2004.00818.x 15566381

[45] Kokkinaki T. Fathers’ speech to infants. Early Child Dev Care. (2013) 183(7):1005–25. 10.1080/03004430.2012.712041

[46] Aspland H Gardner F. Observational measures of parent-child interaction: an introductory review. Child Adolesc Ment Health. (2003) 8(3):136–43. 10.1111/1475-3588.00061 32797579

[47] Smith M. Measures for assessing parenting in research and practice. Child Adolesc Ment Health. (2011) 16(3):158–66. 10.1111/j.1475-3588.2010.00585.x 32847235

[48] Bögels SM van Melick M. The relationship between child-report, parent self-report, and partner report of perceived parental rearing behaviors and anxiety in children and parents. Pers Individ Dif. (2004) 37(8):1583–96. 10.1016/j.paid.2004.02.014

[49] Morsbach SK Prinz RJ. Understanding and improving the validity of self-report of parenting. Clin Child Fam Psychol Rev. (2006) 9(1):1–21. 10.1007/s10567006-0001-5 16636897

[50] Waylen A Stallard N Stewart-Brown S. Parenting and health in mid-childhood: a longitudinal study. Eur J Public Health. (2008) 18(3):300–5. 10.1093/eurpub/ckm131 18202085

[51] Zaslow MJ Weinfield NS Gallagher M Hair EC Ogawa JR Egeland B Longitudinal prediction of child outcomes from differing measures of parenting in a low-income sample. Dev Psychol. (2006) 42(1):27–37. 10.1037/00121649.42.1.27 16420116

[52] Lee R Skinner A Bornstein MH Radford AN Campbell A Graham K Through babies’ eyes: practical and theoretical considerations of using wearable technology to measure parent–infant behaviour from the mothers’ and infants’ view points. Infant Behav Dev. (2017) 47:62–71. 10.1016/j.infbeh.2017.02.006 28347907 PMC5429397

[53] Boyd A Golding J Macleod J Lawlor DA Fraser A Henderson J Cohort profile: the ‘children of the 90s’: the index offspring of the avon longitudinal study of parents and children (ALSPAC). Int J Epidemiol. (2013) 42:111–27. 10.1093/ije/dys064 22507743 PMC3600618

[54] Fraser A Macdonald-Wallis C Tilling K Boyd A Golding J Davey Smith G Cohort profile: the avon longitudinal study of parents and children: ALSPAC mothers cohort. Int J Epidemiol. (2013) 42:97–110. 10.1093/ije/dys066 22507742 PMC3600619

[55] Lawlor DA Lewcock M Rena-Jones L Rollings C Yip V Smith D The second generation of the avon longitudinal study of parents and children (ALSPAC-G2): a cohort profile. Wellcome Open Res. (2019) 4:36. 10.12688/wellcomeopenres.15087.2 31984238 PMC6971848

[56] Northstone K Lewcock M Groom A Boyd A Macleod J Timpson N The avon longitudinal study of parents and children (ALSPAC): an update on the enrolled sample of index children in 2019. Wellcome Open Res. (2019) 4:51. 10.12688/wellcomeopenres.15132.1 31020050 PMC6464058

[57] Harris PA Taylor R Thielke R Payne J Gonzalez N Conde JG. Research electronic data capture (REDCap): a metadata-driven methodology and workflow process for providing translational research informatics support. J Biomed Inform. (2009) 42(2):377–81. 10.1016/j.jbi.2008.08.010 18929686 PMC2700030

[58] Costantini I Cordero M Campbell A Burgess R Glen K Moraitopoulou G Mental Health Intergenerational Transmission (MHINT) Process Manual (2021). 10.31219/osf.io/s6n4h

[59] Sugden NA Mohamed-Ali MI Moulson MC. I spy with my little eye: typical, daily exposure to faces documented from a first-person infant perspective. Dev Psychobiol. (2014) 56(2):249–61. 10.1002/dev.21183 24285109 PMC4262075

[60] Cox JL Holden JM Sagovsky R. Detection of postnatal depression: development of the 10-item edinburgh postnatal depression scale. Br J Psychiatry. (1987) 150(6):782–6. 10.1192/bjp.150.6.782 3651732

[61] Gibson J McKenzie-McHarg K Shakespeare J Price J Gray R. A systematic review of studies validating the Edinburgh Postnatal Depression Scale in antepartum and postpartum women. Acta Psychiatr Scand. (2009) 119(5):350-64. 10.1111/j.1600-0447.2009.01363.x 19298573

[62] Matthey S Barnett B Kavanagh DJ Howie P. Validation of the edinburgh postnatal depression scale for men, and comparison of item endorsement with their partners. J Affect Disord. (2001) 64(2–3):175–84. 10.1016/S0165-0327(00)00236-6 11313084

[63] Meins E Fernyhough C. Mind-mindedness coding manual, Version 2.2. Unpublished manuscript. York, UK: University of York (2015).

[64] Noldus LPJJ. The Observer: A software system for collection and analysis of observational data. Behav Res Meth Instrum Comput. (1991) 23(3):415-29.

[65] Ambady N Rosenthal R. Thin slices of expressive behavior as predictors of interpersonal consequences: a meta-analysis. Psychol Bull. (1992) 111(2):256–74. 10.1037/0033-2909.111.2.256

[66] Burgess R Costantini I Bornstein MH Campbell A Cordero Vega MA Culpin I A Quantitative Evaluation of Thin Slice Sampling for Parent-Infant Interactions. J Nonverbal Behav. (2023) 47(2):117–210. 10.1007/s10919-022-00420-7 37162792 PMC10163135

[67] Kokkinaki T Vasdekis VGS. A study of cognitive (attentional focus) and mentalizing comments of maternal speech: relationship to infant birth order. Int J Psychol Res. (2016) 11(2):145–89.

[68] Bornstein MH Putnick DL Suwalsky JT Gini M. Maternal chronological age, prenatal and perinatal history, social support, and parenting of infants. Child Dev. (2006) 77(4):875–92. 10.1111/j.1467-8624.2006.00908.x 16942495 PMC5827934

[69] Netsi E Pearson RM Murray L Cooper P Craske MG Stein A. Association of persistent and severe postnatal depression with child outcomes. JAMA Psychiatry. (2018) 75(3):247–53. 10.1001/jamapsychiatry.2017.4363 29387878 PMC5885957

[70] StataCorp LP. Stata base reference manual. College Station: StataCorp LLC (2005).

[71] Faul F Erdfelder E Lang A-G Buchner A. G*power 3: a flexible statistical power analysis program for the social, behavioral, and biomedical sciences. Behav Res Methods. (2007) 39(2):175–91. 10.3758/BF03193146 17695343

[72] Littleboy K. Birth characteristics in England and Wales: 2018. London, UK: Office for National Statistics (2019). Available at: https://www.ons.gov.uk/releases/birthcharacteristicsenglandandwales2018

[73] Matthey S. Are we overpathologising motherhood? J Affect Disord. (2010) 120(1-3):263-6. 10.1016/j.jad.2009.05.004. 19628282

[74] Kornhaber M Marcos H. Young children's communication with mothers and fathers: functions and contents. Br J Dev Psychol. (2000) 18(2):187-210. 10.1348/026151000165643

[75] Rowe ML Coker D Pan BA. A comparison of fathers' and mothers' talk to toddlers in low-income families. Soc Dev. (2004) 13(2):278-91.

[76] Hossain Z Roopnarine JL. African-American fathers’ involvement with infants: relationship to their functioning style, support, education, and income. Infant Behav Dev. (1994) 17(2):175–84. 10.1016/0163-6383(94)90053-1

[77] Soderstrom M. Beyond babytalk: re-evaluating the nature and content of speech input to preverbal infants. Dev Rev. (2007) 27(4):501–32. 10.1016/j.dr.2007.06.002

[78] Labrell F. A typical interaction behaviour between fathers and toddlers: teasing. Early Dev Parent. (1994) 3(2):125–30. 10.1002/edp.2430030209

[79] Belsky J Gilstrap B Rovine M. The Pennsylvania infant and family development project, I: stability and change in mother-infant and father-infant interaction in a family setting at one, three, and nine months. Child Dev. (1984) 55(3):692–705. 10.2307/1130122 6734311

[80] Cabrera NJ Volling BL. VIII. Moving research on fathering and children’s development forward: priorities and recommendations for the future. Adv Res Meas Father Child Dev. (2019) 107:7–160. 10.1111/mono.12404

[81] Roggman L Boyce L Cook G Christiansen K Jones D. Playing with daddy: social toy play, early head start, and developmental outcomes. Fathering. (2004) 2(1):83-108. 10.3149/fth.0201.83

[82] Bögels S Phares V. Fathers’ role in the etiology, prevention and treatment of child anxiety: a review and new model. Clin Psychol Rev. (2008) 28:539–58. 10.1016/j.cpr.2007.07.01 17854963

[83] Grossmann K Grossmann KE Fremmer-Bombik E Kindler H Scheuerer-Englisch H Zimmermann P. The uniqueness of the child-father attachment relationship: fathers’ sensitive and challenging play as a pivotal variable in a 16-year longitudinal study. Soc Dev. (2002) 11(3):307–31. 10.1111/1467-9507.00202

[84] Paquette D. Theorizing the father-child relationship: mechanisms and developmental outcomes. Hum Dev. (2004) 47:193–219. 10.1159/000078723

[85] Volling BL McElwain NL Notaro PC Herrera C. Parents’ emotional availability and infant emotional competence: predictors of parent-infant attachment and emerging selfregulation. J Fam Psychol. (2002) 16(4):447. 10.1037/0893-3200.16.4.447 12561291

[86] Abraham E Feldman R. The neural basis of human fatherhood: a unique biocultural perspective on plasticity of brain and behavior. Clin Child Fam Psychol Rev. (2022) 25:93–109. 10.1007/s10567-022-00381-9 35122559

[87] Karberg E Cabrera N Malin J Kuhns C. Chapter VI: longitudinal contributions of maternal and paternal intrusive behaviors to children’s sociability and sustained attention at prekindergarten. Monogr Soc Res Child Dev. (2019) 84(1):79. 33005062 PMC7526692

[88] Lundy BL. Father- and mother-infant face-to-face interactions: differences in mind-related comments and infant attachment? Infant Behav Dev. (2003) 26(2):200–12. 10.1016/S0163-6383(03)00017-1

[89] Rondal JA. Fathers' and mothers' speech in early language development. J Child Lang. (1980) 7(2): 353–69. 10.1017/S0305000900002671 7410499

[90] Paulson JF Dauber S Leiferman JA. Individual and combined effects of postpartum depression in mothers and fathers on parenting behavior. Pediatrics. (2006) 118(2):659–68. 10.1542/peds.2005-2948 16882821

[91] Cummings ME Keller PS Davies PT. Towards a family process model of maternal and paternal depressive symptoms: exploring multiple relations with child and family functioning. J Child Psychol Psychiatry. (2005) 46(5):479–89. 10.1111/j.1469-7610.2004.00368.x 15845128

[92] Faircloth C. Intensive parenting and the expansion of parenting. In: Lee E, Bristow J, Faircloth C, Macvarish J, editors, Parenting culture studies. London: Palgrave Macmillan (2014). p. 25–50.

[93] Hoffman DM. Risky investments: parenting and the production of the ‘resilient child’. Health Risk Soc. (2010) 12(4):385–94. 10.1080/13698571003789716

[94] Shirani F Henwood K Coltart C. Meeting the challenges of intensive parenting culture: gender, risk management and the moral parent. Sociology. (2012) 46(1):25-40. 10.1177/0038038511416169

[95] Vincent C Ball SJ. Making up'the middle-class child: families, activities and class dispositions. Sociology. (2007) 41(6):1061–77. 10.1177/0038038507082315

[96] Dermott E Pomati M. ‘Good’parenting practices: how important are poverty, education and time pressure? Sociology. (2016) 50(1):125–42. 10.1177/0038038514560260

[97] Macvarish J. The problem with neuroparenting. In: Neuroparenting. London: Palgrave Pivot (2016). 10.1057/978-1-137-54733-0_6

[98] Miller T. Making sense of parenthood: Caring, gender and family lives. London: Cambridge University Press (2017).

[99] Gillies V. Teaching and learning guide for: childrearing, class and the new politics of parenting. Sociol Compass. (2009) 3(2):341–4. 10.1111/j.1751-9020.2008.00181.x

[100] Cowan PA Cowan CP. Developmental psychopathology from family systems and family risk factors perspectives: implications for family research, practice, and policy. In: Cicchetti D Cohen DJ, editors. Developmental psychopathology. New York: Wiley (2006). 10.1002/9780470939383.ch14

[101] Dermott E. Contemporary Fatherhood: Intensive or Intimate? Presentation at ESRC Changing Parenting Culture Seminar Series. Seminar 2, Gender and Parenting Culture: Intensive Fatherhood? (2009).

[102] Dermott E Miller T. More than the sum of its parts? Contemporary fatherhood policy, practice and discourse. Fam Relatsh Soc. (2015) 4:183–95. 10.1332/204674315X14212269138324

[103] Lee TTL. Social class, intensive parenting norms and parental values for children. Curr Sociol. (2021):1–18. 10.1177/00113921211048531

[104] Hoff E Laursen B Tardif T. Socioeconomic status and parenting. In: Bornstein MH, editors. Handbookof parenting. 2nd ed. Mahwah, NJ: Erlbaum (2002). p. 231–52.

[105] Masten AS Monn AR. Child and family resilience: a call for integrated science, practice, and professional training. Fam Relat. (2015) 64(1):5–21. 10.1111/fare.12103

[106] Lewis AJ Bertino MD Bailey CM Skewes J Lubman DI Toumbourou JW. Depression and suicidal behavior in adolescents: a multi-informant and multi-methods approach to diagnostic classification. Front Psychol. (2014) 5:766. 10.3389/fpsyg.2014.00766 25101031 PMC4101965

[107] Bardack S Herbers JE Obradović J. Unique contributions of dynamic versus global measures of parent–child interaction quality in predicting school adjustment. J Fam Psychol. (2017) 31(6):649–58. 10.1037/fam0000296 28277709

[108] Chorney JML Gorodzinsky A. Direct observational methods. In: Hopkins B Geangu E Linkenauger S, editors. The Cambridge encyclopedia of child development. 2nd ed. Cambridge: Cambridge University Press (2017). p. 137–40.

[109] Chorney JM McMurtry CM Chambers CT Bakeman R. Developing and modifying behavioral coding schemes in pediatric psychology: a practical guide. J Pediatr Psychol. (2015) 40(1):154–64. 10.1093/jpepsy/jsu099 25416837 PMC4288308

[110] Tschan F Zimmermann J Semmer NK. Rules for coding scheme development. In: Brauner E Boos M Kolbe M, editors. The Cambridge handbook of group interaction analysis, Cambridge handbooks in psychology. Cambridge: Cambridge University Press (2018). p. 191–207.

[111] Pearson RM Heron J Melotti R Joinson C Stein A Ramchandani PG The association between observed non-verbal maternal responses at 12 months and later infant development at 18 months and IQ at 4 years: a longitudinal study. Infant Behav Dev. (2011) 34(4):525–33. 10.1016/j.infbeh.2011.07.003 21840603

[112] Raviv T Kessenich M Morrison FJ. A mediational model of the association between socioeconomic status and three-year-old language abilities: the role of parenting factors. Early Child Res Q. (2004) 19(4):528–47. 10.1016/j.ecresq.2004.10.007

[113] Mills-Koonce WR Propper CB Gariepy JL Blair C Garrett-Peters P Cox MJ. Bidirectional genetic and environmental influences on mother and child behavior: the family system as the unit of analyses. Dev Psychopathol. (2007) 19(4):1073–87. 10.1017/S0954579407000545 17931435

[114] Bornstein MH. Toward a model of culture ↔ parent ↔ child transactions. In: Sameroff A, editors. The transactional model of development: How children and contexts shape each other. Washington, DC: American Psychological Association (2009). p. 139–61.

[115] Sameroff A. The transactional model of development: How children and contexts shape each other. Washington, DV: American Psychological Association (2009).

[116] Xerxa Y Rescorla LA van der Ende J Hillegers MH Verhulst FC Tiemeier H. From parent to child to parent: associations between parent and offspring psychopathology. Child Dev. (2020) 92(1):291–307. 10.1111/cdev.13402 32845015 PMC7891374

[117] Mchale JP. When infants grow up in multiperson relationship systems. Infant Ment Health J. (2007) 28(4):370–92. 10.1002/imhj.20142 21512615 PMC3079566

[118] McHale JP Favez N Fivaz-Depeursinge E. The lausanne trilogue play paradigm: breaking discoveries in family process and therapy. J Child Fam Stud. (2018) 27(10):30633072. 10.1007/s10826-018-1209-y

